# Environmental shocks and migration among a climate-vulnerable population in Bangladesh

**DOI:** 10.1007/s11111-025-00478-7

**Published:** 2025-01-22

**Authors:** Jan Freihardt

**Affiliations:** Center for Comparative and International Studies (CIS), ETH Zurich, 8092 Zurich, Switzerland

**Keywords:** Environmental migration, Riverbank erosion, Flood, Survey, Bangladesh

## Abstract

Various studies predict large migration flows due to climatic and other environmental changes, yet the ex post empirical evidence for such migration is inconclusive. To examine the causal link between environmental changes and migration for a population residing along the Jamuna River in Bangladesh, an area heavily affected by floods and riverbank erosion, I relate the respondents’ self-reported affectedness by environmental changes, their migration aspirations, and their capability to move to their migration likelihood. The analysis relies on a unique quasi-experimental research design based on original survey panel data of 1604 household heads. I find that erosion significantly and substantively increases the likelihood to migrate, leading to more than a doubling of the migration likelihood compared to the unaffected control group. Flooding has a significant effect only if it causes severe and irreversible impacts. Moreover, erosion affectedness increases the likelihood of moving permanently, with the whole household, and to a rural location. Individual, temporary moves to urban locations, by contrast, are primarily driven by low socio-economic status. Those who move with the whole household migrate mostly less than five kilometers. These findings call for a more nuanced understanding of the complex environment-migration nexus.

**Supplementary Information:**

The online version contains supplementary material available at 10.1007/s11111-025-00478-7.

## Introduction

Anthropogenic climate change increases the intensity, duration, and frequency of many hydrological, meteorological, climatological, and biological hazards (IPCC, 2018, 2021; Stott et al., 2016). Two hazards that are of particular relevance due to the large number of people projected to be displaced are floods (Kakinuma et al., 2020; Kam et al., 2021) and sea-level-rise-related coastal erosion (Hauer et al., 2020; Wrathall et al., 2019). Displacement projections are typically based on the number of people projected to live in regions at risk of climate change impacts. Thereby, they deterministically assume that all these people will be forced to move once the climate change impacts materialize. In situations that present an immediate danger to life, people might indeed need to “flee to save their lives” (Warner, 2010, p. 405). In many other cases, however, people might implement technological and/or cultural adaptation strategies that allow them to remain in situ despite climatic changes materializing. Evidence from different contexts suggests that indeed, people prefer adapting in situ over relocating if they have a choice (Amakrane et al., 2023; Jamero et al., 2017; Mallick et al., 2020; Seebauer & Winkler, 2020). To better incorporate such dynamics into models projecting future climate-induced movements, we need a thorough understanding of people’s migration decision-making in response to environmental changes.

The present study contributes to improving our understanding of climate-induced migration by presenting rigorous micro-level evidence on the causal link between environmental changes and migration for a highly vulnerable region in Bangladesh, one of the most climate-change-exposed countries (Rigaud et al., 2018). Micro-level studies are important given that a significant share of the empirical evidence gathered so far is derived from aggregated levels of analysis, i.e., the country or provincial level (Bohra-Mishra et al., 2017; Chen & Mueller, 2019; Feng et al., 2010; Hoffmann et al., 2020; Missirian & Schlenker, 2017; Thiede et al., 2016), which bears the danger of ecological fallacy: Even if, for instance, inter-provincial migration increases in the aftermath of a drought, this does not necessarily mean that the drought was the primary reason motivating people to move (Piguet, 2010; Robinson, 1950).

**Fig. 1:**
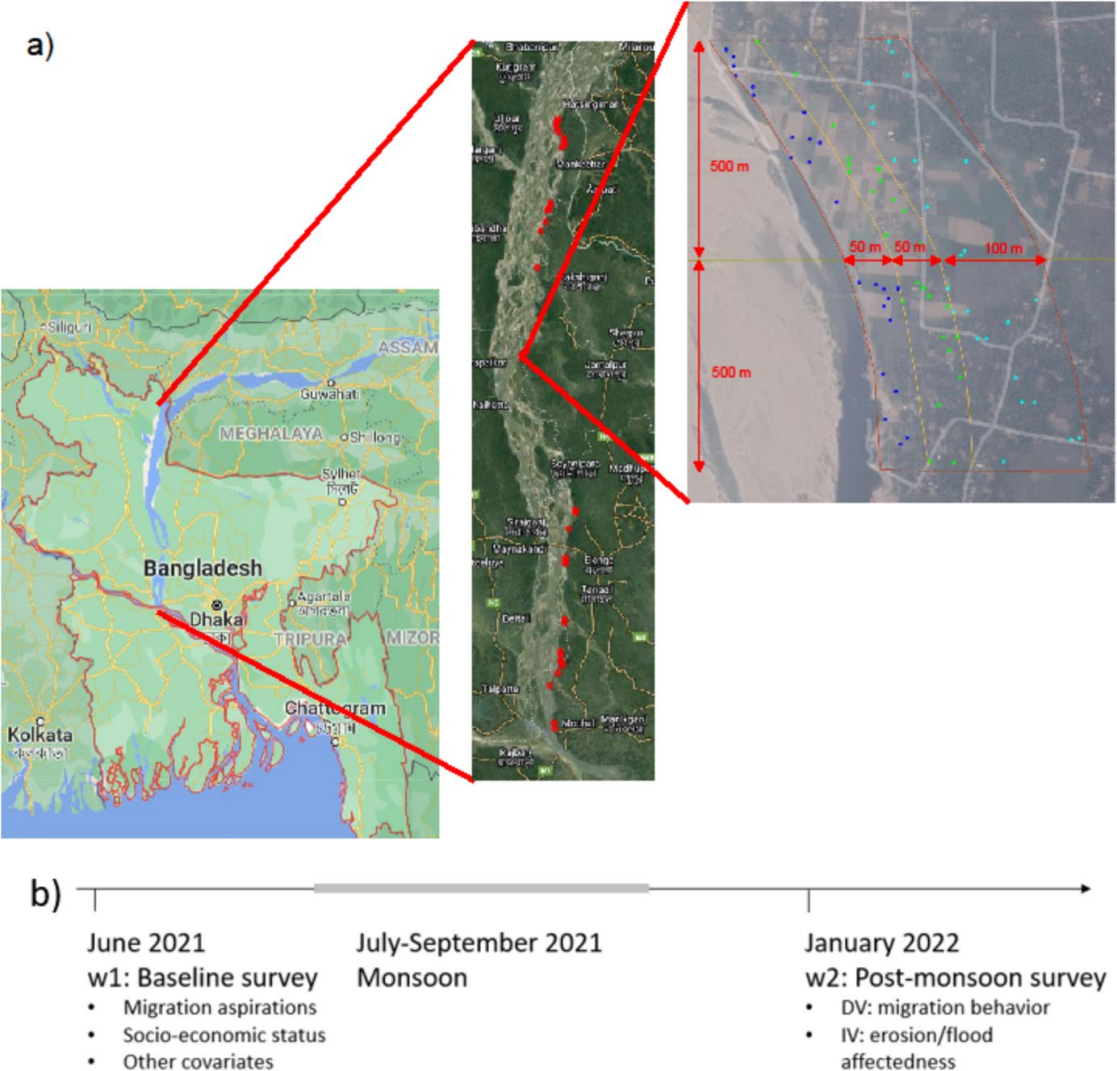
**a** Overview of the Jamuna River, the 36 study locations, as well as the three sampling zones within each location. The study area covered the districts of Manikganj, Tangail, Jamalpur, Sirajganj, Bogra, and Rowmari. Copyright map: Google. Copyright satellite image: TerraMetrics, 2022. **b** Timeline of survey waves and the monsoon season, and overview of which variables were assessed in which wave

The main strength of my approach lies in the unique, first of its kind quasi-experimental research design that I employ. It is based on a large-N, individual-level panel dataset comprising 1604 household heads living in 36 villages along the Jamuna River, one of the three major streams of Bangladesh (Fig. 1a). I interviewed this population at risk of environmental changes both before and after these changes materialized, and re-interviewed both those who migrated and those who remained after the environmental event occurred. This represents a significant improvement over existing micro-level studies which typically interview both non-migrants and migrants only cross-sectionally and retrospectively after an environmental shock has materialized (e.g., Afifi et al., 2016; Islam et al., 2020; Joarder & Miller, 2013; Koubi et al., 2016a, 2016b). In particular, I assessed the respondents’ migration aspirations (i.e., whether they prefer migrating to staying put) and their socio-economic status before the environmental change occurred, and can hence include these potential core confounders into my causal models relating environmental shocks to migration behavior.

As a second contribution, the analysis considers two different types of environmental events, namely riverbank erosion and flooding. The literature proposes that different types of environmental events affect migration behavior differently (Koubi et al., 2016a, 2016b). While flooding and its effects on human migration have been studied extensively (Hirabayashi et al., 2013; Kakinuma et al., 2020; Kam et al., 2021), erosion processes remain relatively understudied. Erosion—meaning that soil gets carried away by water—can occur either gradually in small amounts or abruptly in large quantities (mass failure). The latter is of especially high societal relevance, given that mass failure events can erode agricultural land, and/or destroy houses and infrastructure such as roads within short time. In Bangladesh alone, erosion affects several hundred thousand people each year. Such mass failure events are the focus of this study, rather than slow-onset gradual erosion. While the present study deals with riverbank erosion, yet its findings are likewise relevant for areas exposed to coastal erosion, given that the underlying mechanism (soil being carried away by water) is identical (van Rijn, 2011). As a third contribution, this paper investigates not only the binary decision (migrate vs. stay) but likewise the mode (whole-household vs. individual), destination (international vs. internal, rural-to-urban vs. rural-to-rural), and distance of the observed moves. This enables a more nuanced understanding of the environmental migration dynamics.

## Environmental change and migration

To theoretically explain the link between environmental changes and migration, early environmental migration scholars drew on a neo-Malthusian argument: Environmental change deprives people of their livelihood and therefore leads to migration (de Sherbinin et al., 2022). While such a simple push logic might be appropriate for certain types of irreversible environmental or climatic changes (such as sea-level rise inundating entire island nations or desertification), it appears to be too deterministic for most other types of changes. In particular, most people see migration not as the first best strategy since they are tied to their location and/or are constrained by the economic as well as social/emotional costs of moving (Adams & Kay, 2019; Schewel, 2020). Hence, when confronted with environmental changes, affected people typically first try to adapt in situ to decrease their personal exposure and vulnerability (unless they hold migration aspirations which are unrelated to the environmental changes, for instance, due to educational or labor migration motives). Only once the in situ adaptation efforts fail to sustain their livelihoods, they will consider migration as an option (Mallick, 2019; Penning-Rowsell et al., 2013).

As such, migration decision-making is influenced by a variety of factors extending beyond the mere push logic of environmental stress. Black et al. (2011) propose a conceptual framework for the link between environmental change and migration that encompasses macro- (such as the political system or economic situation of the country), meso- (such as social networks or legal frameworks), and micro-level factors (such as age, gender, or education). Therein, environmental change can influence migration decision-making either directly by affecting ecosystem services or hazard exposure. Likewise, it can have indirect effects by affecting other factors such as the economic system or a household’s assets.

Accordingly, the relationship between environmental change and migration has to be seen in a more nuanced way. Specifically, I draw upon two main theoretical frameworks: First, the aspirations-capabilities framework posits that people migrate if they possess both *aspirations* to migrate and the *capability* to realize this move (Carling, 2002; Carling & Collins, 2018; Carling & Schewel, 2018; de Haas, 2021). Migration aspirations are “a general evaluation of whether or not migrating would be better than staying” (Carling & Schewel, 2018, p. 948). Capabilities relate to the “freedom to choose where to live, including the option to stay” (de Haas, 2021, p. 2). The capability to move can be constrained by various factors, ranging from individual-level (such as health or gender) over household-level (such as wealth or social networks) to meso-level factors (such as migration policies, which are especially relevant for international migration).

Secondly, I follow the findings of the Foresight Project (2011) which stresses the importance of considering a household’s “vulnerability to environmental changes.” While vulnerability is inversely related to wealth, other factors such as education or occupation also play a role: On average, people whose income depends on the environment (such as farmers) can be considered more vulnerable to environmental changes than those who do not depend on the environment (such as teachers). Likewise, more educated people might have more options to change their income source if environmental change deprives them of their livelihood.

These two examples suggest that environmental changes are likely to be perceived differently by different people, depending on their characteristics. Indeed, the literature has found that migration behavior is influenced by individual perceptions of environmental change rather than by objectively identified environmental stressors (Koubi et al., 2016a, 2016b).

Combining the aspirations-capabilities model and the Foresight model, I posit that the link between environmental changes and migration depends on (a) a household’s “vulnerability” to environmental changes, understood as a combination of exposure, sensitivity, and adaptive capacity (IPCC, 2007); (b) a person’s “migration aspirations,” that is a conviction that moving is preferable to staying; and (c) their “capability to move,” that is resources and networks necessary to undertake a move.

**Fig. 2:**
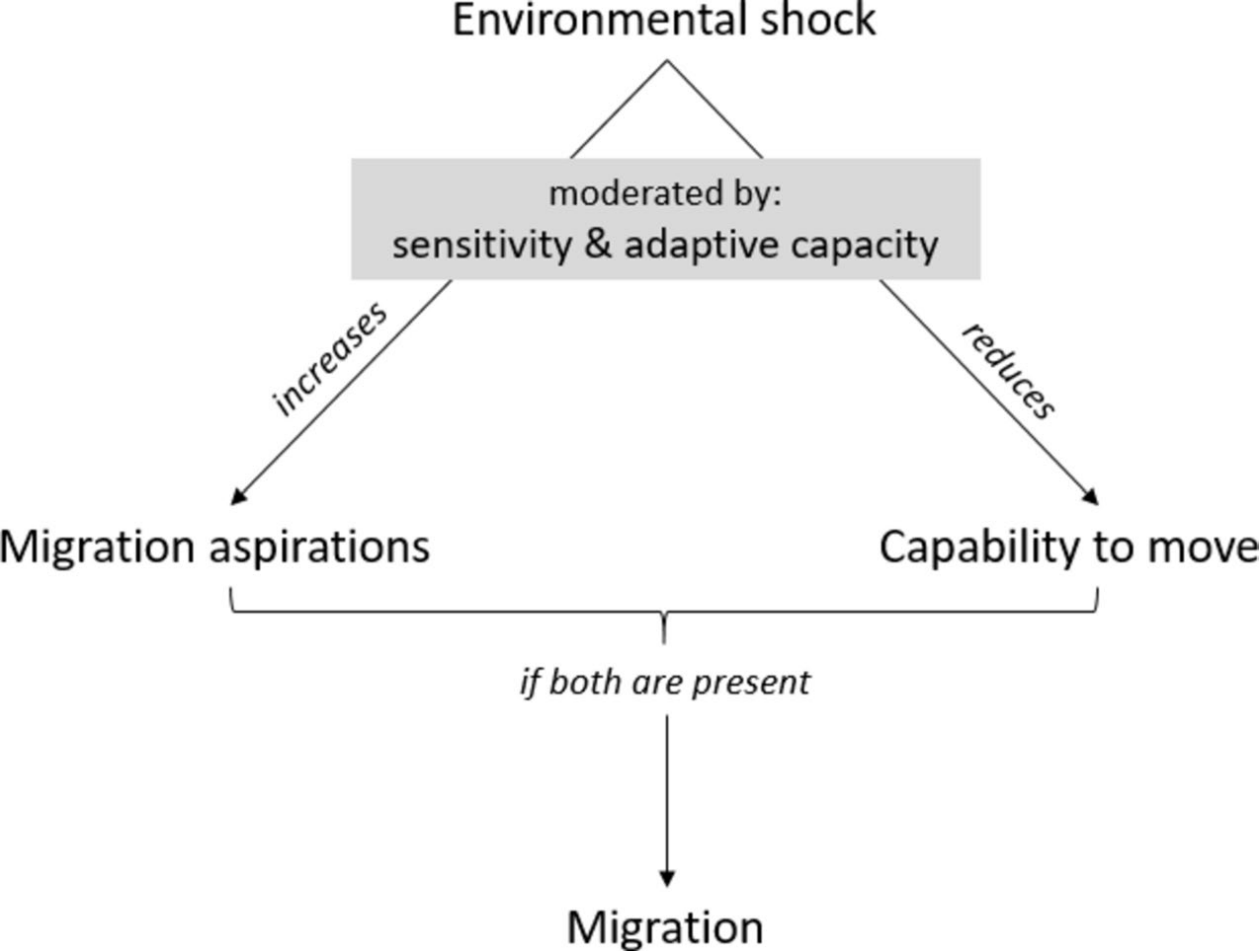
Influence of environmental shocks on migration aspirations and capabilities and, in turn, on migration

As illustrated in Fig. 2, being affected negatively by an environmental shock might increase a person’s migration aspirations. This might for instance be the case if the affectedness leads to a loss of livelihood options, inducing a wish to move elsewhere. Koubi et al. (2022a) analyze the link between environmental changes and migration aspirations for the populations residing along the Jamuna River. Such increased migration aspirations might, in turn, lead to a higher likelihood to move, resulting in the following hypothesis (abbreviated in the following as H):

- *H1a: People affected negatively by environmental changes are more likely to move than those who are not affected.*

Conversely, environmental shocks might reduce a household’s capability to move, for instance, by eroding assets necessary to undertake a move. If this is the case, we might observe a lower likelihood to move among affected populations, leading to the competing hypothesis:

- *H1b: People affected negatively by environmental changes are less likely to move than those who are not affected.*

Besides the factors determining whether affected individuals move or not, the recent environmental migration literature has increasingly focused on differentiating the impact of environmental change on migration by event type (Black et al., 2013; Cattaneo et al., 2019; Koubi et al., 2022b; McLeman, 2014). In particular, it distinguishes between sudden/short-term and gradual/long-term environmental changes.

Sudden/short-term events such as floods or storms can have strong impacts on affected individuals, ranging from casualties over property damage to large-scale economic disruption (Wallemacq & House, 2018). Hence, such events are readily perceived as extreme and might make people move to save their lives or property. Gradual/slow-onset events such as salinization or droughts are typically perceived as less extreme due to their slow-moving, creeping nature. Hence, they do not impose an immediate “need to move” in order to save lives or assets. In addition, given that such events unfold over years or even decades, in situ adaptation might be possible to cope with their adverse effects. In Bangladesh, for instance, farmers change their crop varieties to cope with droughts (Al-Amin et al., 2019) or use sandbags and vegetation to protect riverbanks from eroding (Mamun et al., 2022). Accordingly, I argue:

- *H2: Sudden/short-term events are more likely to lead to migration than gradual/slow-onset events.*

Besides the speed of onset and the duration—the two most important characteristics used to classify environmental events—I posit that one also needs to consider the nature of the impact that an event inflicts on affected households. This is especially important to understand the effect of environmental changes on the distance and duration of the move. Sudden-onset/short-term events typically allow affected people to rebuild their livelihoods once the immediate danger is over. Accordingly, it can be expected that migration in the aftermath of sudden events is short-distance and temporary. These expectations have been confirmed empirically in various contexts (Cattaneo et al., 2019; McLeman & Gemenne, 2018). Gradual changes might lead to migration once such changes have deteriorated the environmental conditions to a point where livelihood strategies are made fully impossible (e.g., if a person’s agricultural land has been fully destroyed). In such a situation, migration can be expected to be more permanent than after sudden-onset events, given that there is little reason to return to the original location. Since gradual changes such as droughts or salinization typically affect larger areas, people need to move further to restore their livelihoods, and hence I expect such moves to be long-distance.

- *H3a: Sudden/short-term events lead to short-distance and temporary moves.*
- *H3b: Gradual events lead to long-distance and permanent moves.*

Given the strong interrelation of environmental shocks, migration aspirations, and the capability to move, it is important to consider interaction effects. With respect to migration aspirations, I follow the theory of planned behavior which posits that intentions are a central factor to perform a given behavior (Ajzen, 1991). Hence, I see attitudes towards a behavior as an important predictor of the actual behavior and expect:

- *H4: Individuals who had migration aspirations prior to the environmental event are more likely to move in the aftermath of the event than those who had no aspirations.*

Concerning the capability to move, one can argue in two ways: On the one hand, individuals with a higher capability to move (which usually corresponds to higher socio-economic status, higher education, etc.) have more options to undertake a move after they have been affected by an environmental event (Gray & Mueller, 2012; Massey & Espinosa, 1997). On the other hand, a higher capability to *move* can also coincide with a higher capability to *adapt* in situ. For example, educated individuals might be able to change their occupation more easily, while wealthy individuals have more means to repair damages they might have incurred (Afifi et al., 2016; Penning-Rowsell et al., 2013). Hence, I posit the competing hypotheses:

- *H5a: Individuals with a higher capability to move are more likely to move in the aftermath of the event than individuals with a lower capability to move.*
- *H5b: Individuals with a higher capability to move are less likely to move in the aftermath of the event than individuals with a lower capability to move.*

## The case: Jamuna River in Bangladesh

Bangladesh is a prime case to study environmental migration. Migration plays an important role in the country’s culture. In terms of international migration, Bangladesh is the sixth-largest migrant sending country in the world, with over seven million Bangladeshi migrants living abroad (IOM, 2024). Likewise, internal migration is an important income diversification strategy for many households. Migration rates that have been observed in different natural hazard-prone areas range from 36% for seasonal migration (Bryan et al., 2014) over 40% for at least one permanent move in the respondents’ lifetime among the eastern riverbank population of the Jamuna—the survey location of this study—(Ferdous et al., 2018) to 95% for temporary migration during the flood season (Paul & Routray, 2010). With respect to future migration flows, the World Bank’s Groundswell report estimates that up to 13.3 million people could be internally displaced by climatic changes until 2050 (Rigaud et al., 2018).

In terms of environmental changes, Bangladesh is among the countries most susceptible to the adverse effects of climate change, due to its topography and its location in one of the largest river deltas of the world (Rigaud et al., 2018). It is affected heavily by sea level rise, frequent cyclones, and high monsoon rainfall that increases river flow, which in turn contributes to extensive flooding and riverbank erosion (Hasan et al., 2018; Islam et al., 2021). All these environmental events will intensify in the future (IPCC, 2018, 2021) and will adversely affect people and their livelihoods by damaging their homesteads and agricultural land, potentially causing economic and social disruptions resulting in large migration flows (Rigaud et al., 2018). In Bangladesh, riverbank erosion and floods are the most impactful processes in terms of yearly damage (Ahmed, 2015).

**Fig. 3:**
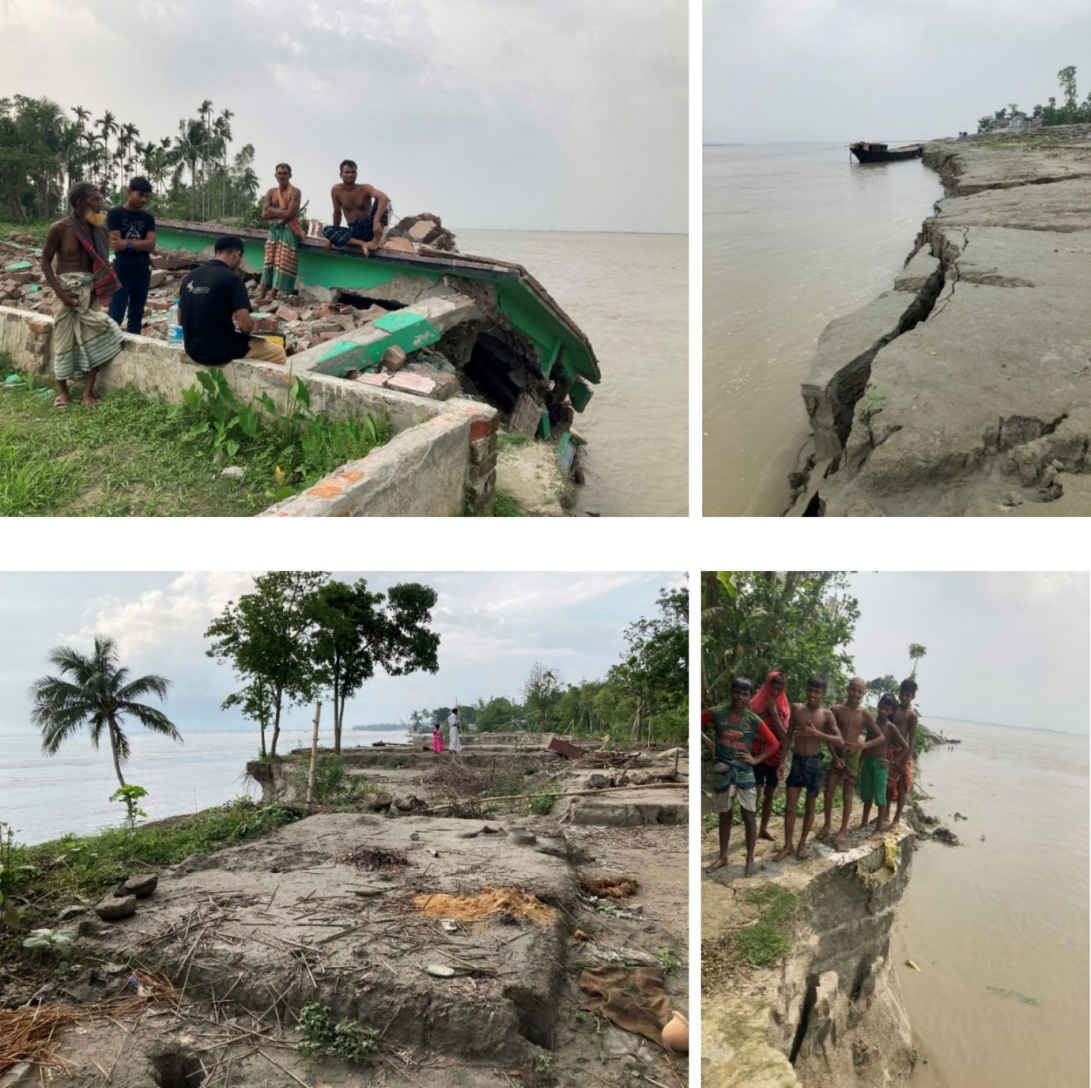
Illustration of erosion along Jamuna River. Photos taken by author during data collection

Regarding riverbank erosion, around 20 out of 64 districts in the country are prone to erosion, which consumes around 8700 ha of land each year and thereby affects around 200,000 people (Alam, 2017). While communities along the rivers are aware of erosion risks, yet people choose to settle next to rivers due to the high soil fertility and/or lack of other suitable space given the country’s high population density. Erosion has several negative impacts on affected communities, including destruction of farmable land, housing, and infrastructure such as roads or schools (see Fig. 3 for a photographic illustration of erosion). Along the 220-km-long Jamuna River, the case region of this study, net erosion was about 933 km^2^ during the 1973–2017 period (CEGIS, 2018a). This would correspond to a widening of the river of more than 4 km if the erosion was distributed evenly along the river. In certain areas, erosion causes an inland shift of the riverbank by several hundred meters per year and often occurs several years in a row. Erosion events occur mainly during the rainy monsoon season, typically from June to October. Since the river erodes more soil than it can transport, a part of the eroded soil is deposited downstream and forms new land in the form of islands (so-called chars). However, the river erodes approximately seven times more land than it forms (Sarker et al., 2014).

Regarding floods, they also occur mostly during the monsoon period, when water levels of the Jamuna River rise to a point where most land—and accordingly, most villages—on its riverbanks are flooded. This regular flooding has an important function in the livelihood cycle of riverine populations, providing humidity and nutrients for their agricultural plots. Only when a flood becomes too severe—either because the water rises too high or because it stays too long—does it turn into a disaster which damages crops, houses, or infrastructure (Alam, 1990). Riverbank erosion and flooding are closely linked, given that severe erosion typically occurs when the force of the flowing water increases shortly before and during the monsoon season.

The impact of floods and erosion is not uniform along and across the river, but varies depending on local natural and anthropogenic characteristics such as elevation or embankments (Ferdous et al., 2018). These characteristics vary not only in space, but also in time, given that the Jamuna River is among the most quickly widening and changing river systems in the world (Oberhagemann et al., 2020). Lastly, variation also occurs between households in their ability to cope with environmental change. The populations residing next to the rivers have developed a range of coping strategies to minimize the damage resulting from floods (e.g., raising the platform of the house; Paul & Routray, 2010) and erosion (e.g., planting vegetation on the riverbank; Mamun et al., 2022). Since coping strategies are usually related to socio-economic status, not all households are equally capable of adopting these strategies. This significant variation between as well as within villages forms the basis for the causal inference design employed in this study, which is introduced in the following sections.

## Materials and methods

### Survey overview

For the empirical analysis, this study uses data from two waves of a panel survey among 1604 household heads from 36 locations distributed along the whole length of the Jamuna River in Bangladesh (Fig. 1a). The baseline survey (wave 1) was conducted in June and July 2021 before the onset of the monsoon season during which floods and erosion occur.

Participants were selected in a multi-stage cluster design with the goal of making the sampled population representative of the population at risk of riverbank erosion. In the first stage, 36 locations were selected along the easternmost riverbank line of the Jamuna because the rates of riverbank erosion are higher along the eastern than along the western riverbank due to differences in floodplain materials (CEGIS, 2018b; Sarker et al., 2014). This line was identified using the most recent satellite imagery available. Villages on chars (sandy islands in the river) were not considered since char populations have adapted their livelihoods to the yearly recurring flood and erosion events (Alam, 2017; Islam et al., 2015).

Along this line, 250 sampling points were defined with a 1-km distance along the whole stretch of the river (from the border with India in the north to the convergence of Ganges and Jamuna in the south). Ideally, the 36 study locations would have been drawn randomly from this pool of 250 stretches. However, a visual, satellite-based analysis revealed that not all of these 250 stretches were suitable for the study purpose. Therefore, each of the 250 stretches was evaluated with respect to the following two criteria: First, survey feasibility, that is, whether there were enough settlements (= at least 75 houses) in the 200-m stretch inland; and second, the ex ante risk for riverbank erosion, in particular, whether there was a clear indication of a permanent embankment structure that prevents erosion and whether there was a char/large sandbank in front of the stretch that blocks erosion.

Stretches for which the satellite analysis showed that at least one of these criteria was violated were excluded from the pool. This reduced the pool size from 250 to 79 stretches. For some of these 79 stretches, not all criteria could be clearly evaluated from the satellite images due to insufficient image resolution. Therefore, the final screening was done on the ground during a field visit. Six stretches could not be visited due to their remote location. Of the remaining 73 stretches, 29 were excluded after the field visit due to a violation of at least one of the three criteria. One stretch was used for training the enumerators, leaving 43 stretches suitable for the sample. Due to time constraints during the fieldwork, not all 43 stretches could be included in the sample. Therefore, 36 stretches were chosen such that they were well distributed along the entire length of the Jamuna. An overview of the 36 locations is provided in Fig. 1a. Table S3 provides a list of all 79 stretches initially in the sample, including whether they were part of the final sample and—if not—the reason for their exclusion.

At each of the 36 locations, households were sampled using a stratified random spatial sampling design to survey households located within three zones defined by distance from the shoreline. This design allowed capturing potential effects of different ex ante erosion risk levels on perceptions of environmental changes. At each location, the three zones were constructed by shifting the shoreline inland by 50 m, 100 m, and 200 m, respectively. Consequently, each sampling zone has an extent of 1 km in the direction of flow and of 200-m inland.

Within each of the three zones, a spatially explicit sample was generated following the procedure outlined by Crawford et al. (2020). Specifically, a set of 25 random latitude–longitude points per zone was created using ArcMap software (with a minimum distance of 10 m between points). In the field, enumerators navigated to these points using smartphones. Having arrived at the point, they selected the house closest to that point based on visual estimation. This household was subsequently interviewed (see Fig. S4 for the distribution of the households’ distance from the riverbank). If a household declined participation or if the household head was not available at two contact attempts, the enumerator continued to the next closest household, in reference to the starting point. Within each household, the household head was interviewed, defined as the decision-maker within the household.

Between wave 1 and the post-monsoon survey (wave 2), contact with respondents was maintained via regular phone calls to ensure being able to find and re-interview them after the monsoon. Wave 2 was conducted between January and March 2022 among both affected and unaffected respondents, and among both migrants and non-migrants (Fig. 1b). Panel attrition between the two waves was less than 6% and was higher for migrants (19%) than for non-migrants (4%), since migrants were more frequently not able to make time for the interview due to job obligations (Table S4). Migrants were interviewed in their new location or via phone.

The respondents are 88% male, 92% married, on average 47 years old, and have mostly no or only primary education (Table S5). Fifty-six percent of the respondents depend on the environment for their primary income source, either by working on their own or others’ agricultural land. Other common income sources are owning a small business/shop, non-agriculture related day labor, remittances, transport, and textile weaving. Interviews were conducted face-to-face in Bangla by native interviewers (students from different Bangladeshi universities trained and supervised in situ by the author) and lasted for about 45–60 min. The questionnaire included both closed and open-ended questions pertaining to respondents’ experience with migration and environmental events as well as personal and household information. The parameters assessed in the questionnaires are summarized in Table S6. Respondents received a monetary compensation for the time spent on the survey. The study including the full questionnaire has been pre-registered at OSF (Freihardt et al., 2021); details are provided in Appendix F.

### Operationalization

The migration behavior of all respondents was recorded in the post-monsoon survey (see Fig. 1b for an overview of which variables are taken from which survey wave). Thereby, migration is defined as moving to a location outside of the administrative boundary of the village. From this, four different outcome variables are constructed to capture respondents’ migration behavior: First, a binary indicator of *migration status*, taking a value of 1 if the respondent has undertaken any move between wave 1 and wave 2, and a value of 0 for non-migrants who have not left the village between the two survey waves. Hence, this includes whole-household as well as individual moves. Additional models presented in the robustness section model (a) only whole-household moves and (b) only individual moves. Second, an indicator of *migration destination*, taking a value of 1 if a respondent moved to a rural location (specified in the survey as a village), a value of 2 if she moved to an urban location (specified as a town/city), and a value of 0 if no move was undertaken. Third, an indicator of *migration mode*, taking a value of 1 if a respondent moved individually (meaning that the family stayed in the village), a value of 2 if they moved with the whole household, and 0 if no move was undertaken. Fourth, the *migration distance* of whole-household migrants is calculated from the distance between their house coordinates as recorded in wave 1 and wave 2, respectively.

In terms of independent variables, *environmental shocks* are operationalized by the respondents’ self-reported affectedness by erosion and floods, respectively, during the 2021 monsoon season, rather than relying on objectively measured data. This is important since no two people perceive environmental changes in the exact same way, be it due to demographic (such as age or gender) or socio-economic characteristics (such as the importance of the environment for one’s livelihood (Freihardt, 2024; Koubi et al., 2016a, 2016b; Osbahr et al., 2011). Respondents’ affectedness was assessed in retrospect in wave 2, by asking “Has the 2021 erosion (flood) had an impact on your household?” as well as—for those who had been affected—“What was the impact?”. In the first set of analyses, I use binary indicators of affectedness, taking a value of 1 if a respondent stated to have been personally affected by erosion and floods, respectively. In the second set, I subdivide affectedness by four degrees of severity (Table S7, see Fig. S5 for a descriptive overview of the impact categories).

While it has been shown that environmental perceptions matter to understand migration behavior (Koubi et al., 2016a, 2016b), using self-reported data to operationalize affectedness by environmental shocks might raise the concern of motivated reasoning: Respondents who have left might overstate the erosion/flood impacts to justify their migration behavior. To address this concern, respondents’ subjective assessment of the impact category “Loss of house” (see Table S7) is compared to objective data. Specifically, this analysis uses respondents’ house coordinates as registered during wave 1 and the satellite-based erosion assessment tool developed by Freihardt and Frey (2023) to identify those respondents whose house was eroded during the 2021 monsoon. Overall, self-reported and objective data are coherent for 94% of all respondents, increasing the confidence in relying the main analyses on self-reported impacts. A more detailed discussion is provided in Appendix A. The robustness section presents models which operationalize environmental shocks by this objective indicator of erosion affectedness instead of self-reported impacts, with substantively comparable results.

With respect to the main potential confounders, first, the respondents’ *migration aspirations* are measured by their binary migration aspirations in wave 1, taking a value of 1 if they answered either “yes” to the question “During the last 5 years, have you thought seriously about leaving this village?” or if they answered “Move somewhere else” to the question “Right now, if you could choose, would you stay here in this village or would you prefer to move to another place?”. Second, the respondents’ *capability to move* is proxied by their socio-economic status, given that economic resources are a prerequisite for migration (McLeman & Smit, 2006). Socio-economic status is operationalized by the first component of a principal component analysis (PCA) on asset ownership, assessed in wave 1 (see Table S8 for dimensions/components of the PCA). Note that it is essential to measure aspirations and capability in wave 1 (pre-treatment) as both of them can be influenced by environmental shocks.

Different other covariates were included in the analyses which previous studies have found to be important for migration behavior: In terms of *occupation type*, the respondents’ main income source was recoded as either environmentally independent (0) or environmentally dependent (1). Thereby, environmentally dependent respondents are expected to be more likely to migrate in the aftermath of an environmental event due to the strong link between their livelihood and the respective environmental conditions (de Longueville et al., 2020). *Place attachment* is measured on a 5-point-scale from 1—“not attached at all” to 5—“very attached.” It has been shown that place attachment can have a stronger effect on immobility than resource constraints (Adams, 2016; Adams & Adger, 2013). Respondents self-assessed their *risk preference* on a 5-point-scale from 1—“I never take chances” to 5—“I always take chances.” Risk averse individuals are expected to have a lower likelihood to move since the risk of moving is perceived as higher than the risk of staying (Carling, 2002). Further covariates included in the analysis were the respondents’ age, education, sex, and marital status, since these have been shown to influence migration behavior (Adger et al., 2015; Hunter et al., 2015). Summary statistics and a correlation matrix of all relevant variables are presented in Table S5 and Table S9, respectively. Overall, the variables are only weakly correlated, with the exception of sex and marital status, whereby female sex is correlated to an unmarried status.

### Estimation strategy

An extensive description of the estimation strategy is provided in Appendix C.1. In short, the study aims at establishing a causal link between environmental changes and migration behavior by defining a treatment group (respondents who have been affected by environmental changes) and a control group (unaffected respondents). Assuming that both groups have on average comparable characteristics for all parameters that might influence the outcome variable (i.e., migration behavior), any differences in migration behavior that are observed between the two groups can be attributed to the fact that one group has been affected by environmental changes and the other one has not. In other words: Other common migration reasons such as economic (e.g., unemployment) or social reasons (e.g., education or marriage) are assumed to be equally prevalent in treatment and control group and need hence not be modeled explicitly. This assumption is tested by balance tests between treatment and control group (see Appendix B). To improve the credibility of a causal interpretation of the model results, the results section presents additional models which include (a) entropy balancing weights and (b) covariates that could correlate with erosion/flood exposure. Lastly, to investigate the interplay of environmental shocks, migration aspirations, and the capability to move, interaction models are estimated between flood/erosion affectedness and migration aspirations and socio-economic status, respectively.

## Results

### Impact of flood/erosion affectedness on migration likelihood

**Fig. 4:**
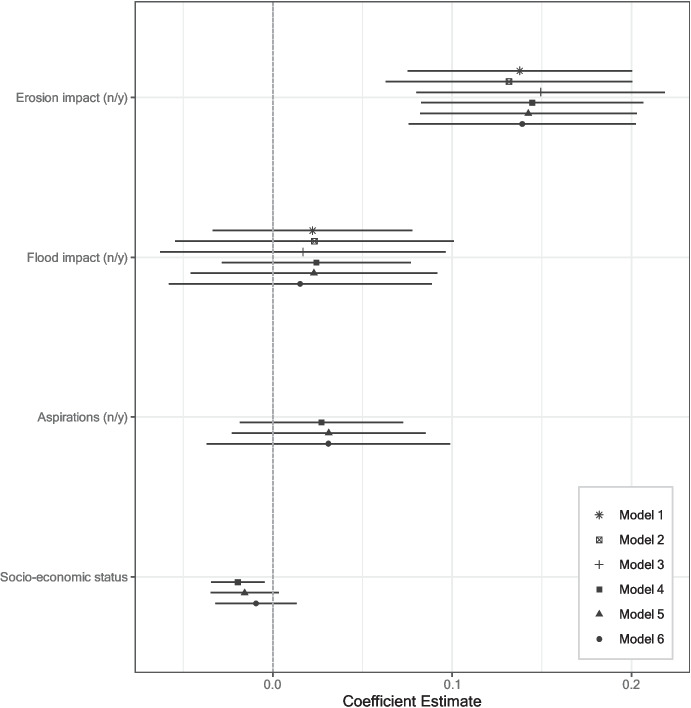
Influence of binary flood and erosion affectedness on whether a respondent migrated between wave 1 and wave 2 (linear regressions). Models 1–3: no controls; models 4–6: including control variables. Models 1/4: no entropy-balancing weights; models 2/5: including entropy-balancing weights based on erosion treatment; models 3/6: including entropy-balancing weights based on flood treatment. Mean migration rate among erosion control group: 0.11. (n/y)—(no/yes). Whiskers correspond to 95% confidence intervals

Figure 4 presents the influence of flood and erosion affectedness on whether respondents undertook any move between wave 1 and wave 2. Model 1 contains direct effects without controls and entropy balancing weights, while models 2 to 6 present robustness checks by including entropy balancing weights (models 2, 3, 5, and 6) and control variables (models 4 to 6), respectively (see Table S10 for full model results).

Consistently across models, having been affected by erosion in the previous monsoon season significantly increases the likelihood of a respondent to move in between wave 1 and wave 2, lending evidence to hypothesis H1a. This effect is also substantively relevant: Respondents who were affected by erosion are between 13 and 15 percentage points more likely to undertake a move than those who were not affected. This corresponds to more than a doubling of the migration likelihood, given the control group mean of 11 percentage points (see statistics in Table S10). Having been affected by a flood does not on average increase the migration likelihood to a significant extent. This finding hence contradicts hypothesis H2, expecting floods to have a stronger effect on migration than erosion.

Models 4 to 6 control for migration aspirations and socio-economic status (as a proxy for the capability to move). The effect of migration aspirations does not reach conventional levels of significance. Socio-economic status is inversely related to migration likelihood, meaning that wealthier respondents are less likely to move than respondents with less resources.

In terms of the remaining covariates (Table S10), male and younger respondents are more likely to migrate than female and older respondents, respectively. These effects are in line with previous literature findings (Hunter et al., 2015). Marital status, education, income source, place attachment, and risk preference do not exhibit significant effects on the migration likelihood. Respondents who live further from the riverbank are significantly less likely to undertake a move, while respondents from the district of Kurigram are significantly more likely to move. To sum up, erosion affectedness significantly increases the migration likelihood, while flood impacts remain insignificant.

### Interaction effects

**Fig. 5:**
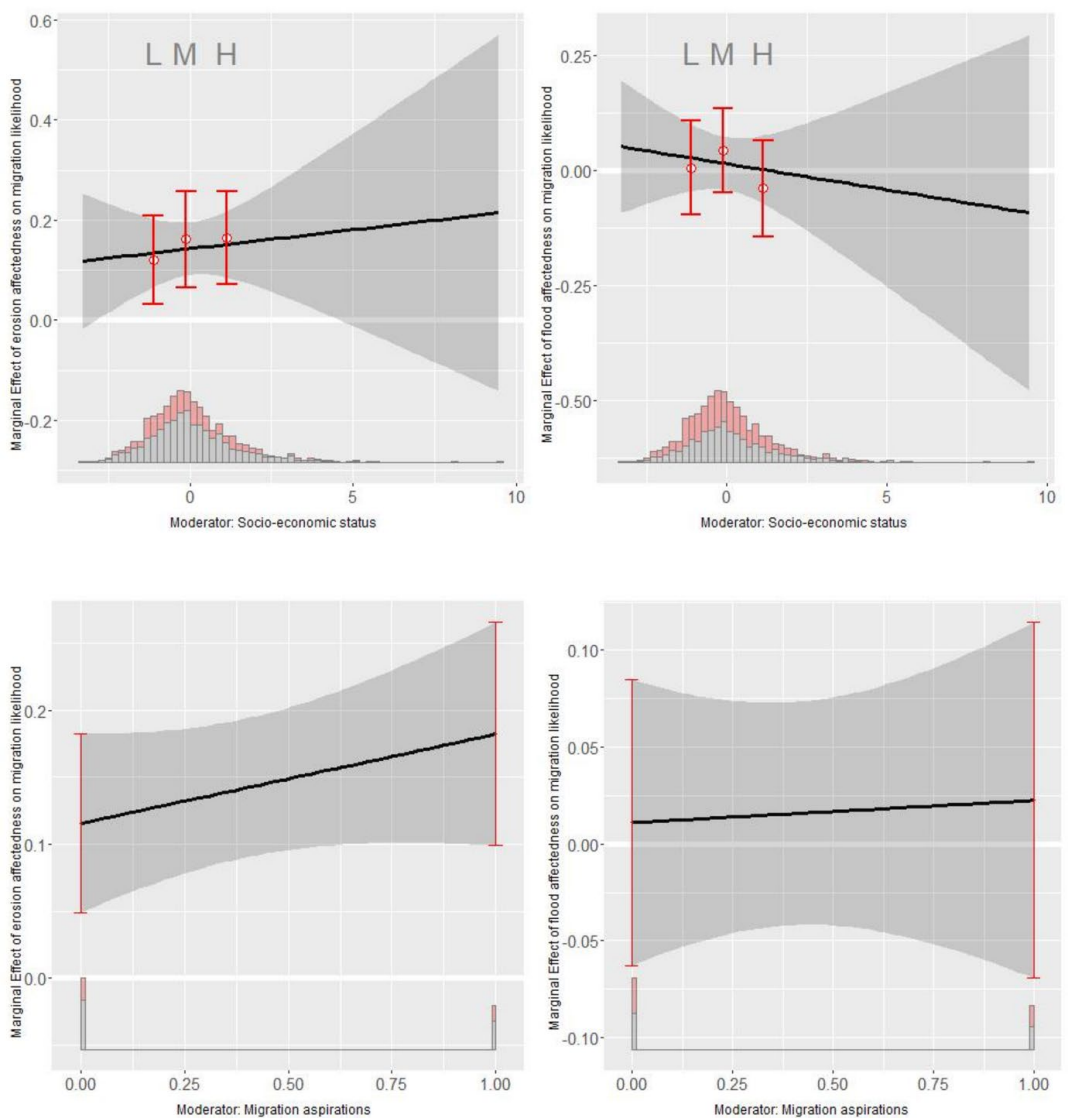
Marginal effects plot of the interaction between erosion (left column) and flood affectedness (right column) and socio-economic status (upper row) and migration aspirations (lower row). The model setup corresponds to model 5 from Table S10 (including entropy balancing weights and controls)

So far, I have considered only the direct effects of flood/erosion affectedness on the migration likelihood. However, and as I argued above, migration occurs if migration aspirations and the capability to migrate concur. Hence, I also analyze interactive models (Hainmueller et al., 2019). Specifically, I am interested in how the effect of flood/erosion affectedness on the migration likelihood changes with differing levels of socio-economic status and migration aspirations (Fig. 5). For erosion affectedness, I observe a significant positive relationship with socio-economic status: the higher an individual’s socio-economic status, the stronger the effect of erosion affectedness on their migration likelihood. This suggests that households with lower socio-economic status might have less means to migrate after having been affected by erosion than wealthier households and, hence, risk becoming “trapped,” thus supporting hypothesis H5a. Likewise, in line with hypothesis H4, the relationship with migration aspirations is positive—erosion affectedness has a stronger effect on individuals who possessed migration aspirations prior to the environmental shock than on those who did not have any aspirations. For flood affectedness, the effects of socio-economic status and aspirations are not significantly different from zero. Hence, they do not exert a meaningful moderating effect on the migration likelihood after having been affected by flooding.

### Different degrees of affectedness

**Fig. 6:**
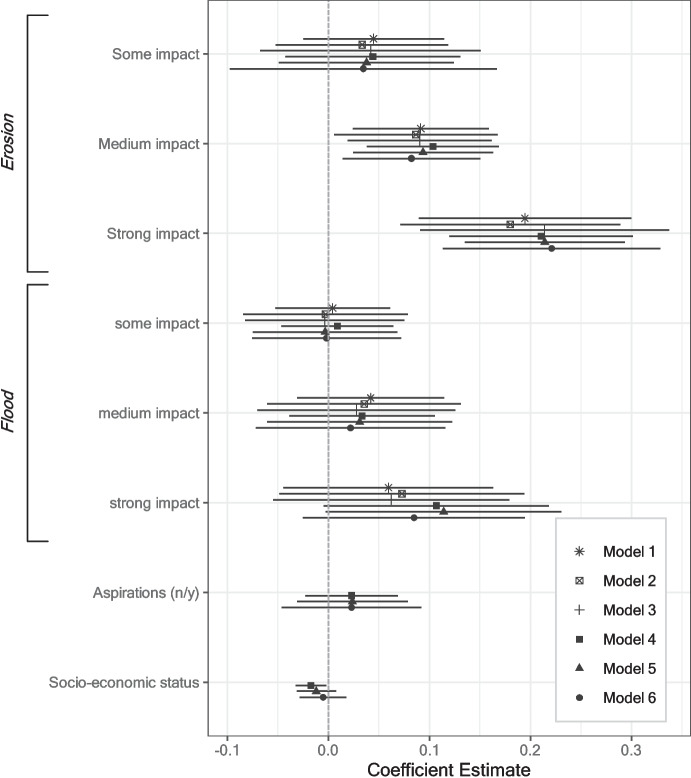
Influence of the severity of flood and erosion affectedness on whether a respondent migrated between wave 1 and wave 2 (linear regressions). Models 1–3: no controls; models 4–6: including control variables. Models 1/4: no entropy-balancing weights; models 2/5: including entropy-balancing weights based on erosion treatment; models 3/6: including entropy-balancing weights based on flood treatment. Mean migration rate among erosion control group: 0.11. Baseline erosion/flood impact: no impact. (n/y)—(no/yes). Whiskers correspond to 95% confidence intervals

The analyses presented above are based on a binary indicator of flood/erosion affectedness. Affectedness can, however, take different forms that could influence migration differently. For instance, respondents who lose their house due to erosion may *have* to move, while others who only lose part of their crops may perceive a less immediate need to move. To investigate the extent to which results are driven by severe impacts, Fig. 6 displays how different degrees of affectedness by erosion and floods, respectively, influence respondents’ migration behavior, relative to a baseline of no affectedness. The remaining model set-up remains identical compared to Fig. 3 (see Table S11 for the full model results). For erosion, some impact does not have a significant influence on the migration likelihood, while both medium and strong impacts increase the likelihood to migrate significantly and substantively (with an increase of up to 22 percentage points for strong impacts, corresponding to a tripling compared to the control group which had not experienced any erosion impacts). These findings are robust across model specifications and suggest that it is not only a loss of land or house (which are classified as strong impacts) that makes respondents leave their village. For floods, only strong impacts in models 4 to 6 significantly increase the migration likelihood, albeit to a lower substantive extent than the effects of erosion affectedness (up to 11 percentage points increase).

### Migration destination

**Fig. 7:**
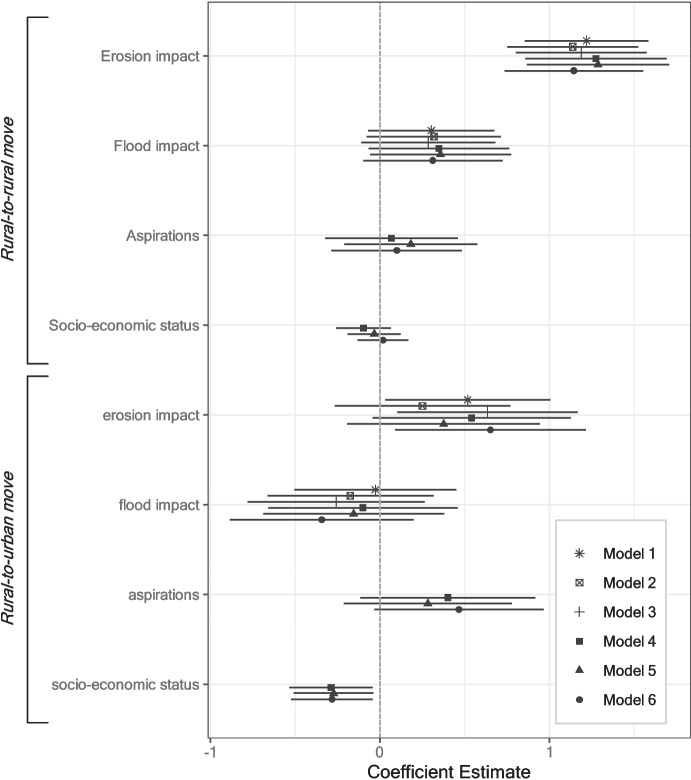
Influence of binary flood and erosion affectedness on migration destination (multinomial logit models). Models 1–3: no controls; models 4–6: including control variables. Models 1/4: no entropy-balancing weights; models 2/5: including entropy-balancing weights based on erosion treatment; models 3/6: including entropy-balancing weights based on flood treatment. Baseline dependent variable: no move. Whiskers correspond to 95% confidence intervals

Besides the binary migration decision, I model where to and for how long respondents migrate. Only six out of 217 migrants (2.7%) moved outside of Bangladesh. This is in line with a large body of literature suggesting that most environmental migration occurs internally (Clement et al., 2021; Rigaud et al., 2018). Ninety-eight percent of all moves are less than 20 km in distance, with 64% of the respondents moving less than 5 km (Fig. S6). Figure 7 presents the results of modeling whether those respondents who stayed within Bangladesh move to a rural or to an urban area (with a baseline of no move; see Table S12 for full model results). Rural-to-rural moves are significantly more likely if a respondent is affected by erosion. Rural-to-urban moves are also more likely for erosion-affected respondents. However, this effect is substantively less pronounced than for rural-to-rural moves, and not fully robust to the inclusion of weights or controls. It hence appears that erosion-affected households predominantly move to other rural areas. Staying in rural contexts is primarily motivated by the option of continuing agriculture as well as by maintaining social ties to the home village. The exact destination is then often determined by the availability of housing (typically with relatives) and agricultural land (an extremely scarce resource in Bangladesh). Flood impacts do not exert a consistently significant influence on either type of move. Lastly, the lower the respondents’ socio-economic status, the more likely they are to move to an urban location.

### Migration mode and duration

**Fig. 8:**
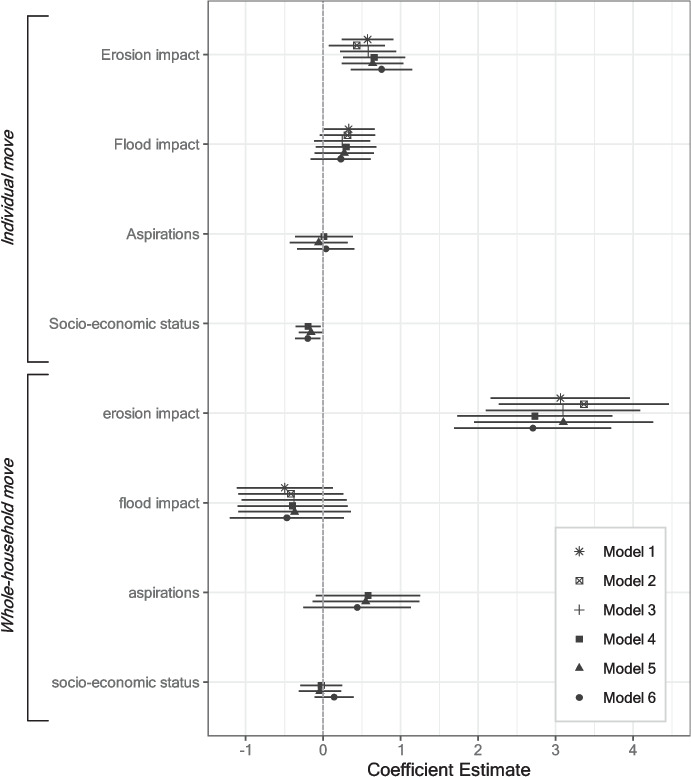
Influence of binary flood and erosion affectedness on migration mode (multinomial logit models). Models 1–3: no controls; models 4–6: including control variables. Models 1/4: no entropy-balancing weights; models 2/5: including entropy-balancing weights based on erosion treatment; models 3/6: including entropy-balancing weights based on flood treatment. Baseline dependent variable: no move. Whiskers correspond to 95% confidence intervals

In terms of migration mode, Fig. 8 presents the results of modeling whether respondents move individually or with the whole household (with a baseline of no move; see Table S13 for full model results). Individual moves are more likely for respondents who are affected by erosion and who have a lower socio-economic status, compared to erosion-unaffected respondents and those with higher socio-economic status, respectively. For whole-household moves, only affectedness by erosion exhibits a significant effect which is substantively much stronger than for individual moves. Whereas 61% of the individual migrants plan to return to their home village, this is the case only for 21% of the whole-household migrants. Individual moves can hence mostly be considered temporary, and whole-household moves mostly permanent. This finding thus supports hypothesis H3b. Flood affectedness does not influence the migration mode to any significant extent, contrary to hypothesis H3a.

### Robustness tests

The robustness section Appendix E in the Supplementary Information provides evidence on four aspects: (a) logistic instead of linear regressions; (b) considering village-level affectedness; (c) different specifications of the dependent variable (analyzing whole-household and individual migration separately; considering also migration of household members; and considering also shifting of the household to another location within the village); and (d) different specifications of the independent variables (modeling only erosion/only flooding/a combined indicator; and employing an objective, satellite-derived indicator of house loss instead of the subjective self-assessment of affectedness). Overall, these tests support the main finding of a strong link between erosion affectedness and migration, and a weak or non-existent relationship between flood affectedness and migration.

## Discussion

This study contributes to furthering our understanding of the environment-migration nexus in three ways. First, it establishes a credible causal link between environmental changes and an increased migration likelihood among the population residing along the Jamuna River in Bangladesh—hence contradicting the hypothesis that environmental changes might decrease the migration likelihood. In particular, affectedness by erosion strongly increases the migration likelihood, while flood affectedness has an overall insignificant effect. The latter finding is in line with recent studies from Bangladesh reporting that flooding has an insignificant or even negative effect on migration (Call et al., 2017; Chen et al., 2017; Gray & Mueller, 2012; Chen & Mueller, 2018).

A relevant aspect to explain the less pronounced effect of floods on migration is that unlike erosion, floods play an important role in the livelihood cycle of riverine populations. The communities residing along the Jamuna are largely dependent on agriculture, which in turn requires nutrients and humidity provided by the yearly recurring monsoon flooding. And while the flood season undoubtedly imposes a lot of hardship, given that not only fields are flooded but also the respondents’ villages and houses, adaptation options exist for most of the resulting challenges, such as building an earth foundation to raise one’s house above the flood level or transporting goods by boat instead of via land. Comparable household level adaptation is barely possible for erosion except for deconstructing and shifting one’s house shortly before it gets eroded—a widespread adaptation strategy along the Jamuna (Hutton & Haque, 2004; Mamun et al., 2022; Penning-Rowsell et al., 2013). Nonetheless, I show that it is not only the loss of land or house that leads to migration, but that also medium severe erosion impacts significantly increase the migration likelihood. Besides household level adaptation, also community, NGO, and government support structures are much more developed for flood impacts than for erosion (Brouwer et al., 2007). These findings suggest that it is important to consider the type of impact that an environmental event inflicts on affected individuals to understand the link between environmental changes and migration.

A second noteworthy finding concerns the interplay of socio-economic status and migration in the aftermath of environmental shocks. The literature suggests, alternatively, that wealthier (H5a) or poorer individuals (H5b) are more likely to move after being affected by environmental changes. My findings suggest that households with lower socio-economic status might have less means to migrate after having been affected by erosion than wealthier households. This raises the question whether certain parts of the study population become “trapped” in unfavorable environmental conditions. “Trapped populations” describe involuntary non-migrants, meaning parts of the population who would like to move away, but lack the capacity to do so. While the term has seen a lot of conceptual discussion in recent literature (cf. Ayeb-Karlsson et al., 2018 for a discursive review), more thorough empirical work is required to investigate the extent and nature of trapped populations. Concerning the interaction with migration aspirations, it appears that prior aspirations increase the effect of erosion affectedness on the migration likelihood, underlining the importance of considering aspirations as an important element of the migration decision-making process. Overall, however, it is noteworthy that erosion impacts were a much stronger predictor of migration in wave 2 than aspirations expressed in wave 1.

Third, regarding the respondents’ migration destination and mode, erosion affectedness significantly increases the likelihood for rural-to-rural as well as for whole-household moves. Rural-to-urban as well as individual moves are made more likely by erosion affectedness and by a low socio-economic status. Taken together, these findings suggest that environmentally induced migration in the study area is predominantly rural-to-rural, with the whole household, and mostly permanent. Importantly, this finding contrasts previous literature which has found that climatic changes increase rural-to-urban migration (Ahsan et al., 2011; Barrios et al., 2006; Nawrotzki et al., 2017). In the context of northern Bangladesh, individual, mostly temporary moves to urban contexts seem to be driven by economic motives and might hence constitute an income-diversifying strategy of low-income households, which send one or several household members to work elsewhere while the rest of the household stays behind. Such temporary migration is a common strategy to cope with economic stress in Bangladesh (Call et al., 2017; Martin et al., 2014). Importantly, I find that even those who move with the whole household migrate mostly less than 5 km. This adds to evidence from other studies in Bangladesh which find that disaster-affected people move on average less than two miles (Hutton & Haque, 2004; Islam et al., 2020), less than 5 km (Ferdous et al., 2018) or—more generally—within Bangladesh rather than internationally (Cundill et al., 2021).

Given that this study presents micro-level evidence for one specific case study, it is worthwhile discussing the generalizability of the findings to other settings. The results could be directly relevant for other riverine populations which are affected by riverbank erosion and flooding—two phenomena that occur not only in Bangladesh, but likewise along other major rivers worldwide such as the Nile, the Yellow River, or the Mekong (Kummu et al., 2008; Yao et al., 2011). Looking beyond the specific event types, the finding that the severity of impacts matters for migration decisions might be transferred to other contexts where destruction of housing or land occurs. Examples include sea-level rise, coastal erosion, or storms. Ultimately, however, the generalizability of the findings can only be confirmed by applying the research design of this study to other geographical contexts and to other types of environmental and climatic changes. Likewise, the present study is limited to a one-time environmental shock, whereas many villages are affected repeatedly by erosion and flooding. For instance, the study region was hit by a severe flood in 2020, the year before the first wave of this panel study (Han et al., 2021). Examining the cumulative effect of recurring environmental events is an important avenue for future research.

## Conclusion

In terms of policy relevance, the link between erosion affectedness and migration appears particularly noteworthy. While many respondents who lost their house or land migrated, still the majority stayed in the village even after having suffered such drastic livelihood impacts. Possible explanations include strong social, emotional, and economic ties to the location, substantive adaptive capacities, or lack of the necessary means to move despite the desire to do so. Furthermore, most of the whole-household moves observed are short-distance and rural-to-rural, hence contradicting the narrative that is commonly put forward by the media and certain political actors, namely that environmentally induced migration is primarily long-distance and directed to urban centers or even other countries. Finally, this study reveals a high likelihood to migrate focusing only on one single monsoon season. Considering that erosion occurs each year, thereby affecting several hundred thousand households in Bangladesh, the absolute number of people migrating due to erosion affectedness becomes sizable if several years are considered. In turn, this suggests that when it comes to climate-related migration, supporting both those who move (e.g., by facilitating access to capital), and those who stay in vulnerable regions (e.g., by investing in protective infrastructure and effective relief programs) should feature more prominently on the political agenda in times of rapidly accelerating climatic changes.

## Supplementary Information

Below is the link to the electronic supplementary material.

Supplementary file1 (DOCX 538 KB)

## Data Availability

The data underlying the results is available at 10.5281/zenodo.10444264.
