# Supplementary material for: Environmental shocks and migration among a climate-vulnerable population in Bangladesh
Source: Popul Environ. 2025 Jan 22;47(1):6. doi: 10.1007/s11111-025-00478-7 (PMC11754328; doi:10.1007/s11111-025-00478-7)
Supplement: Supplementary file 1 — Supplementary file1 (DOCX 538 KB) [file 11111_2025_478_MOESM1_ESM.docx]

**Supporting Information for**

Environmental shocks and migration among a climate-vulnerable population in Bangladesh

Published in: Population and Environment

Jan Freihardt^1,*^

^1^Center for Comparative and International Studies (CIS), ETH Zurich, 8092 Zurich, Switzerland

Email: [fjan@ethz.ch](mailto:fjan@ethz.ch)

ORCID: [0000-0003-2096-0335](https://orcid.org/0000-0003-2096-0335)

**This PDF file includes:**

Appendix A: Comparison of objective erosion data to self-reported impacts

Appendix B: Balance checks

Appendix C: Supporting information for Material and Methods section

C.1 Estimation strategy

C.2 Additional figures and tables

Appendix D: Supporting information for Results section

Appendix E: Robustness checks

E.1 Logistic regressions

E.2 Village-level affectedness

E.3 Different specifications of migration (dependent variable)

E.4 Different specifications of environmental affectedness (independent variable)

Appendix F: Pre-registration

F.1 Anonymized version of pre-analysis plan

F.2 Modifications to the pre-analysis plan

SI References

Appendix A: Comparison of objective erosion data to self-reported impacts

A challenge for causally identifying the link between environmental events and migration is that there exist no objective data on exposure. Satellite imagery on flood extent and depth is not available at the household scale, as cloud cover during the monsoon prevents such data from being gathered. Additionally, the Bangladesh government or other organizations do not provide flood maps with the detail required. Satellite imagery on erosion occurrence can be gathered by comparing riverbeds before and after the monsoon (Freihardt & Frey, 2023). However, in both cases, even if we had perfect exposure data on erosion and flood occurrence available, this would not tell us to what extent individual households are affected. For example, flood or erosion of uninhabited land will directly affect households that farm on or own this land – but such objective data cannot be linked to individuals given that individual-specific land-use maps are not available. Hence, I primarily rely on self-reported exposure directly inquired from respondents, which gives a nuanced picture on whether respondents report to have been affected at all, and to what extent. As I rely on straightforward factual questions (“Were you affected by erosion/floods?” and “What was the first, second and third most important impact on your household?”), and as the communicated scientific study goals provide no direct incentives for respondents to under- or overstate affectedness (other than with NGO- or government-sponsored surveys), I am confident that this approach leads to a sincere measure of affectedness. Still, I acknowledge that such self-reported assessments can exhibit biases. For example, it would be particularly worrisome if households that have migrated self-justify the move with flood or erosion impact, and subsequently overstate such impacts.

Therefore, I draw on one objective, and likely accurate measure of an erosion impact, namely the loss of house, to verify A) whether a self-reported loss of house and a satellite-based indication that the GIS location of the respondent’s home was eroded coincide and B) whether regressions of *objective* exposure and migration behavior show a consistent pattern compared to regressions of *subjective* exposure and migration behavior (discussed in the robustness section).

Specifically, I use respondents’ house coordinates as registered during wave 1 and the satellite-based erosion assessment tool developed by Freihardt & Frey (2023) to identify those respondents whose house was eroded during the 2021 monsoon. First, I compare this objective indicator of house loss to whether respondents self-reported any erosion impacts (Table S 1). Out of the 158 respondents who objectively lost their house, 144 (91%) self-reported to have been affected by erosion. The remaining 9% did not report impacts despite the satellite imagery identifying them as having lost their house. Second, I compare the objective data to the more specific impact categories (Table S 2). Out of the 158 respondents who objectively lost their house, 109 (69%) self-reported to have lost their house. Out of 1446 respondents whose house location did not get eroded according to the satellite imagery, 1406 (97%) did not self-report a loss of house. Overall, this means that self-reported and objective data are coherent for 94% of all respondents. 49 respondents (3% of all respondents) indicated no loss of house, despite the satellite analysis revealing that their house location has been eroded. However, considering which specific impacts were indicated by this subset of respondents (Fig. S 1), the majority of them still indicated a severe or medium impact (permanent displacement, total loss of land, damage of the house) – all of which are plausible impacts for the case where their house location has been eroded. The remaining 40 respondents (3% of all respondents) indicated a loss of their house which was not confirmed by the satellite analysis. Several reasons might explain this mismatch: First, respondents might have lost their house in an earlier monsoon season and mistakenly indicated it as an impact of the 2021 monsoon. Second, technical problems (e.g., recording the coordinates or extracting the bank line from satellite imagery) might result in mis-classifications of the satellite analysis. Third, enumerators might have mistakenly clicked the wrong category. Overall, however, these analyses increase my confidence in relying the main analyses on self-reported impacts, given that only 3% of all respondents can be classified as not in line with objective data.

Table S 1: Cross-tabulation of any self-reported erosion impacts and objectively determined house loss.

|  | **Objectively lost their house** | |  |
| --- | --- | --- | --- |
| **Self-reported any erosion impact** | No | Yes | Row Total |
| No | 1064 (66.3%) | 14 (0.9%) | 1078 |
| Yes | 382 (23.8%) | 144 (9.0%) | 526 |
| Column Total | 1446 | 158 | 1604 |

Table S 2: Cross-tabulation of self-reported erosion-induced house loss and objectively determined house loss.

|  | **Objectively lost their house** | |  |
| --- | --- | --- | --- |
| **Self-reported loss of house** | No | Yes | Row Total |
| No | 1406 (87.7%) | 49 (3.1%) | 1455 |
| Yes | 40 (2.5%) | 109 (6.8%) | 149 |
| Column Total | 1446 | 158 | 1604 |


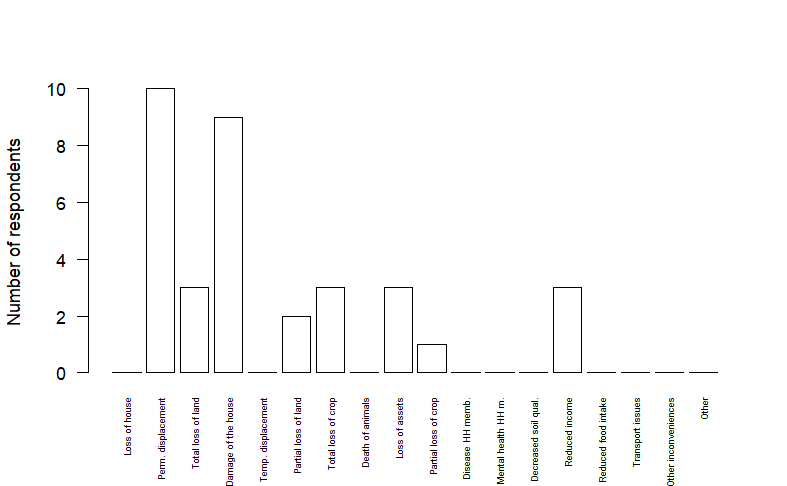


Fig. S 1: Primary erosion impact as self-reported by those respondents who did not indicate to have lost their house, but for whom the satellite-based analysis revealed a house loss.

Appendix B: Balance checks

The core assumption to establish a causal link between environmental changes and migration is exogeneity of the treatment. This means that *ex ante*, treatment and control group should have comparable characteristics on all covariates that might influence the outcome variable (= migration behavior).

To test this assumption, I perform balance checks on relevant covariates: a) spatial factors: district to which the household’s village belongs, distance of the household to the riverbank before the monsoon season (bank_2021); b) socio-demographic factors: education, age, sex; marital status; c) income-related variables: environmental dependence of the primary income source (w1_income_source1_coded), socio-economic status (ses); and d) psychological variables that might influence migration behavior: migration aspirations before the monsoon (w1_aspirations), risk preference and attachment to the village.

Fig. S 2 and Fig. S 3 present the standardized mean difference of these variables for the two treatment variables “household has been affected by erosion” and “household has been affected by flood”, respectively. A standardized mean difference of zero means that treatment and control group are on average equal with regard to the respective variable. The larger the mean difference, the larger the difference between the two groups.

For both erosion and flood treatment, most mean differences are within ±0.1, which indicates good balance and supports my understanding of flood/erosion affectedness as a natural and hence quasi-random process. However, a few exceptions exist, most notably with respect to geospatial factors: In terms of erosion affectedness (Fig. S 2), certain districts are more affected than others, most notably Tangail. Further, respondents who live further away from the riverbank (bank_2021) are on average less affected by erosion. This makes sense, given that those households right next to the riverbank have the highest risk of being affected. In terms of flooding (Fig. S 3), imbalances are less pronounced. For instance, there is no imbalance in flood affectedness by distance to the riverbank (bank_2021) – which again makes sense, given that flooding typically extends several hundred meters or even a few kilometers inland, hence affecting the entire village.

From these analyses, I infer that district and distance to riverbank are potential confounders for which treatment and control groups are not balanced. Hence, I present additional models in the results section including matching of treatment and control groups on the distribution of the variables “district” and “bank_2021”.


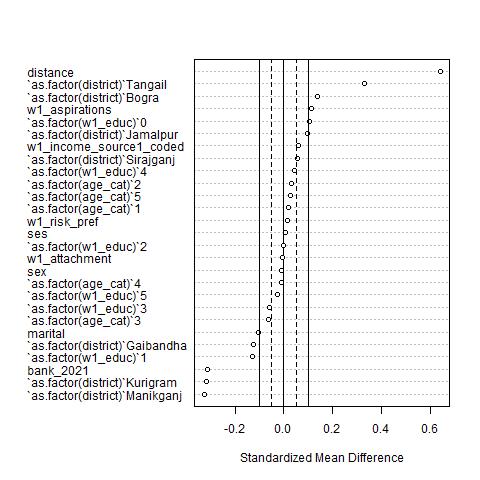


Fig. S 2: Summary of balance between treatment and control group for treatment = "affected by erosion". Note that "distance" in the top-left is a one-dimensional summary of all the included covariates, and not the household’s distance to the riverbank (which is given by the variable “bank_2021”).


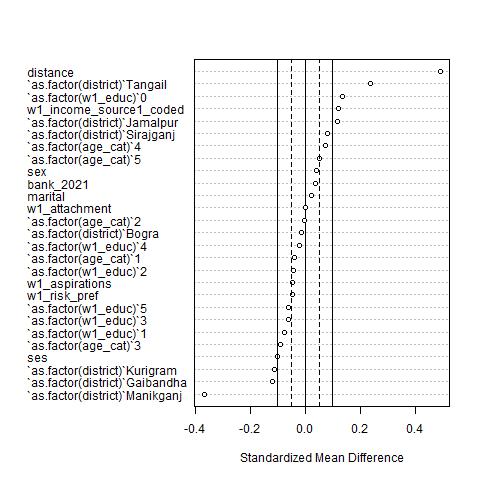


Fig. S 3: Summary of balance between treatment and control group for treatment = "affected by flood". Note that "distance" in the top-left is a one-dimensional summary of all the included covariates, and not the household’s distance to the riverbank (which is given by the variable “bank_2021”).

Appendix C: Supporting information for Material and Methods section

C.1 Estimation strategy

In this paper, I aim at establishing a causal link between environmental changes and migration behavior. Causal inference requires a counterfactual – what would affected respondents have done had they not been affected by the environmental change? To proxy this counterfactual, I define a treatment group (respondents who have been affected by environmental changes) and a control group (unaffected respondents). Given that the assignment into treatment and control group is not controlled like in a lab experiment, but occurs based on natural variation on where floods and erosion materialize, this design constitutes a quasi-experiment.

Assuming that both groups have on average comparable characteristics for all parameters that might influence the outcome variable (i.e., migration behavior), I can attribute any differences in migration behavior that I observe between the two groups to the fact that one group has been affected by environmental changes and the other one has not. Hence, a causal link between environmental changes and migration is possible. Accordingly, I estimate the following baseline models (one for each outcome variable, respectively):

*migration status_i_ / destination_i_ / mode_i_ ~ erosion affectedness_i_ + flood affectedness_i_ + ε_j_*

for respondents i = 1, 2, …, N. For the binary indicator of migration status, I estimate linear probability models, which facilitate the interpretation of the model results since the coefficients of flood/erosion impact can directly be understood as an in-/decrease of the probability to migrate. As robustness checks, I additionally estimate logistic regression models which might be more appropriate for binary outcome variables (Horrace & Oaxaca, 2006). For migration destination and mode, multinomial logit models are applied. Standard errors are clustered at the village level. The model coefficients of erosion and flood affectedness, respectively, can be interpreted as the causal influence of affectedness on the respective migration outcome variable.

This causal interpretation depends on the assumption of control and treatment group having on average comparable characteristics. To test this assumption, I conduct balance tests between treatment and control group (see Appendix B). From these analyses, I infer that most mean differences are within ±0.1 for both erosion and flood treatment, which indicates good balance and supports my understanding of flood/erosion affectedness as a natural and hence quasi-random process. However, a few exceptions exist, most notably with respect to geospatial factors: In terms of erosion affectedness, certain districts are more affected than others. Further, respondents who live further away from the riverbank are on average less affected by erosion. This might imply endogeneity issues, meaning that the affectedness by erosion and floods correlates with pre-treatment population characteristics.

Note that of particular relevance might be an imbalance with respect to socio-economic status, whereby poorer households might *a priori* be more likely to be affected by erosion/flooding than wealthier ones. This might be the case if a geographical sorting occurs, whereby wealthier households move away from the riverbank *before* erosion happens, while poorer households have less options to move and are forced to stay in more vulnerable locations close to the riverbank. Such a geographical sorting would challenge a core assumption required to establish a causal link between environmental changes and migration, namely the random selection into treatment: If poorer households are more likely to be affected, then treatment and control group can no longer be assumed to be on average comparable with respect to socio-economic status. Indeed, socio-economic status and distance to the riverbank are significantly and positively correlated, indicating that wealthier household live on average further away from the riverbank (Table S8). However, the correlation coefficient between socio-economic status and distance to the riverbank is weak, and the balance checks presented in Appendix B reveal no significant imbalance in terms of socio-economic status. This suggests that there are overall no worrisome sorting effects with respect to socio-economic status.

To improve the credibility of a causal interpretation of my model results, I present additional models in the results section. First, to address potential endogeneity issues arising from imbalances between treatment and control group, I match treatment and control group observations on pre-treatment measures of geographic, socio-demographic, and attitudinal variables at the individual and household level. I use entropy balancing (Hainmueller, 2012) to calculate two sets of weights that achieve balance in the distribution of the first moments (mean), second moments (variance) and third moments (skewness) of these covariates for i) the erosion treatment and control group and ii) the flood treatment and control group. This implies that the control groups are weighted such that the respective treatment group’s mean, variance, and skewness is matched on the following pre-treatment variables: migration aspirations, socio-economic status, sex, education, age, marital status, income source, place attachment, risk preference, distance to bankline, district as well as flood/erosion affectedness (the opposite event to the treatment variable on which matching is performed).

Second, I estimate additional models including covariates that could correlate with erosion/flood exposure to address any remaining endogeneity concerns (see section 3.4). These variables were measured pre-treatment in wave 1 (Fig. 1b). This constitutes a significant improvement over cross-sectional studies in which covariates are measured post-treatment. Controlling for post-treatment variables might introduce bias if these variables are a) mediators, meaning that they are on the causal pathway between erosion/flood affectedness and migration (such as household wealth which might be negatively affected by erosion/flood exposure, while in turn also affecting migration behavior), or b) colliders, meaning that they are influenced by both treatment and outcome variable (such as a respondent’s occupation type which might change due to an erosion-related loss or due to moving away from the village).

Lastly, to investigate the interplay of environmental shocks, migration aspirations, and the capability to move, I estimate interaction models between flood/erosion affectedness and migration aspirations and socio-economic status, respectively. These interaction models include entropy balancing weights and controls.

C.2 Additional figures and tables

Table S 3: List of 79 stretches initially in the sample.

| **Site** | **Latitude** | **Longitude** | **District** | **Sampled** | **Reason for exclusion** |
| --- | --- | --- | --- | --- | --- |
| 1 | 23.84858386 | 89.77728293 | Manikganj | Yes |  |
| 2 | 23.85741259 | 89.7761692 | Manikganj |  | Suitable, excluded for time constraints |
| 3 | 23.8659592 | 89.77336891 | Manikganj | Yes |  |
| 4 | 23.8741437 | 89.7696434 | Manikganj |  | Training site |
| 5 | 23.88306306 | 89.76938992 | Manikganj |  | Suitable, excluded for time constraints |
| 6 | 23.89188075 | 89.77113454 | Manikganj |  | Embankment |
| 7 | 23.93977265 | 89.77300493 | Manikganj |  | Not visited |
| 8 | 23.96265272 | 89.75980911 | Manikganj |  | Char/sandbank |
| 9 | 23.971464 | 89.75801152 | Manikganj |  | Char/sandbank |
| 10 | 23.97946232 | 89.76071457 | Manikganj | Yes |  |
| 11 | 23.98646574 | 89.76636763 | Manikganj |  | Not enough settlement |
| 12 | 23.99336663 | 89.77212095 | Manikganj |  | Not enough settlement |
| 13 | 24.00092974 | 89.77700303 | Manikganj |  | Not enough settlement |
| 14 | 24.0080967 | 89.78240531 | Manikganj |  | Not enough settlement |
| 15 | 24.01468743 | 89.78848452 | Tangail | Yes |  |
| 16 | 24.01998107 | 89.79575917 | Tangail |  | Not visited |
| 17 | 24.02782786 | 89.79999907 | Tangail |  | Not visited |
| 18 | 24.03669272 | 89.80143637 | Tangail | Yes |  |
| 19 | 24.04554666 | 89.80018357 | Tangail | Yes |  |
| 20 | 24.05359151 | 89.79620979 | Sirajganj | Yes |  |
| 21 | 24.06191619 | 89.79537304 | Sirajganj |  | Not enough settlement; char/sandbank |
| 22 | 24.07061319 | 89.79705522 | Sirajganj | Yes |  |
| 23 | 24.07936755 | 89.79571167 | Sirajganj | Yes |  |
| 24 | 24.08826802 | 89.79443957 | Sirajganj |  | Not enough settlement; char/sandbank |
| 25 | 24.09651555 | 89.79100124 | Sirajganj |  | Char/sandbank |
| 26 | 24.10416064 | 89.78625988 | Sirajganj |  | Char/sandbank |
| 27 | 24.11252358 | 89.78760715 | Sirajganj |  | Not enough settlement; embankment |
| 28 | 24.1199595 | 89.79245961 | Sirajganj |  | Embankment |
| 29 | 24.12686204 | 89.79824051 | Sirajganj |  | Embankment |
| 30 | 24.13388967 | 89.80382386 | Sirajganj |  | Embankment |
| 31 | 24.14197229 | 89.80777083 | Sirajganj |  | Embankment |
| 32 | 24.15055663 | 89.81028585 | Tangail |  | Not enough settlement; embankment |
| 33 | 24.15953767 | 89.81062441 | Tangail |  | Suitable, excluded for time constraints |
| 34 | 24.16836954 | 89.80947906 | Tangail | Yes |  |
| 35 | 24.17727796 | 89.80949719 | Tangail | Yes |  |
| 36 | 24.2295696 | 89.78659759 | Tangail |  | Not visited |
| 37 | 24.23854823 | 89.7860587 | Tangail |  | Not visited |
| 38 | 24.34224707 | 89.81162957 | Tangail | Yes |  |
| 39 | 24.35117757 | 89.81254109 | Tangail | Yes |  |
| 40 | 24.36017152 | 89.81221315 | Tangail | Yes |  |
| 41 | 24.36889043 | 89.81040512 | Tangail |  | Not enough settlement; embankment |
| 42 | 24.38032687 | 89.8048785 | Tangail |  | Embankment |
| 43 | 24.43313402 | 89.8199736 | Tangail |  | Char/sandbank |
| 44 | 24.44195819 | 89.82141819 | Tangail | Yes |  |
| 45 | 24.45047689 | 89.82397444 | Tangail | Yes |  |
| 46 | 24.49471362 | 89.84519411 | Tangail | Yes |  |
| 47 | 24.50358737 | 89.84590586 | Tangail | Yes |  |
| 48 | 24.51181581 | 89.84245001 | Tangail |  | Embankment |
| 49 | 24.58706697 | 89.81975046 | Jamalpur |  | Not enough settlement; embankment |
| 50 | 24.59501471 | 89.81563181 | Jamalpur |  | Not enough settlement; embankment |
| 51 | 24.60315021 | 89.81184187 | Jamalpur |  | Embankment |
| 52 | 24.61077011 | 89.80728877 | Jamalpur |  | Embankment |
| 53 | 24.61961004 | 89.80620216 | Jamalpur |  | Embankment |
| 54 | 24.9010855 | 89.65554932 | Bogra |  | Not visited |
| 55 | 24.96551006 | 89.66537782 | Bogra | Yes |  |
| 56 | 24.97114163 | 89.67177488 | Bogra | Yes |  |
| 57 | 24.9745598 | 89.68009865 | Jamalpur |  | Not enough settlement |
| 58 | 24.98684177 | 89.69272319 | Jamalpur |  | Not enough settlement |
| 59 | 25.00071823 | 89.70405209 | Jamalpur |  | Char/sandbank |
| 60 | 25.21211443 | 89.72276084 | Jamalpur |  | Not enough settlement |
| 61 | 25.22079034 | 89.7204164 | Jamalpur | Yes |  |
| 62 | 25.22963506 | 89.7196273 | Jamalpur | Yes |  |
| 63 | 25.33554382 | 89.72866088 | Gaibandha | Yes |  |
| 64 | 25.36243599 | 89.7450544 | Jamalpur | Yes |  |
| 65 | 25.38816632 | 89.74946849 | Kurigram |  | Suitable, excluded for time constraints |
| 66 | 25.39617635 | 89.75357157 | Kurigram | Yes |  |
| 67 | 25.40397791 | 89.75805218 | Kurigram | Yes |  |
| 68 | 25.41161802 | 89.76276468 | Kurigram | Yes |  |
| 69 | 25.42006097 | 89.76266573 | Kurigram | Yes |  |
| 70 | 25.58524338 | 89.80014675 | Kurigram | Yes |  |
| 71 | 25.59256635 | 89.80534785 | Kurigram | Yes |  |
| 72 | 25.60067086 | 89.80889396 | Kurigram | Yes |  |
| 73 | 25.60950232 | 89.80727078 | Kurigram | Yes |  |
| 74 | 25.62799682 | 89.78911681 | Kurigram | Yes |  |
| 75 | 25.63403951 | 89.78356735 | Kurigram | Yes |  |
| 76 | 25.64292418 | 89.78384213 | Kurigram | Yes |  |
| 77 | 25.65177817 | 89.78241922 | Kurigram | Yes |  |
| 78 | 25.66053126 | 89.78367557 | Kurigram | Yes |  |
| 79 | 25.66861671 | 89.78750184 | Kurigram | Yes |  |


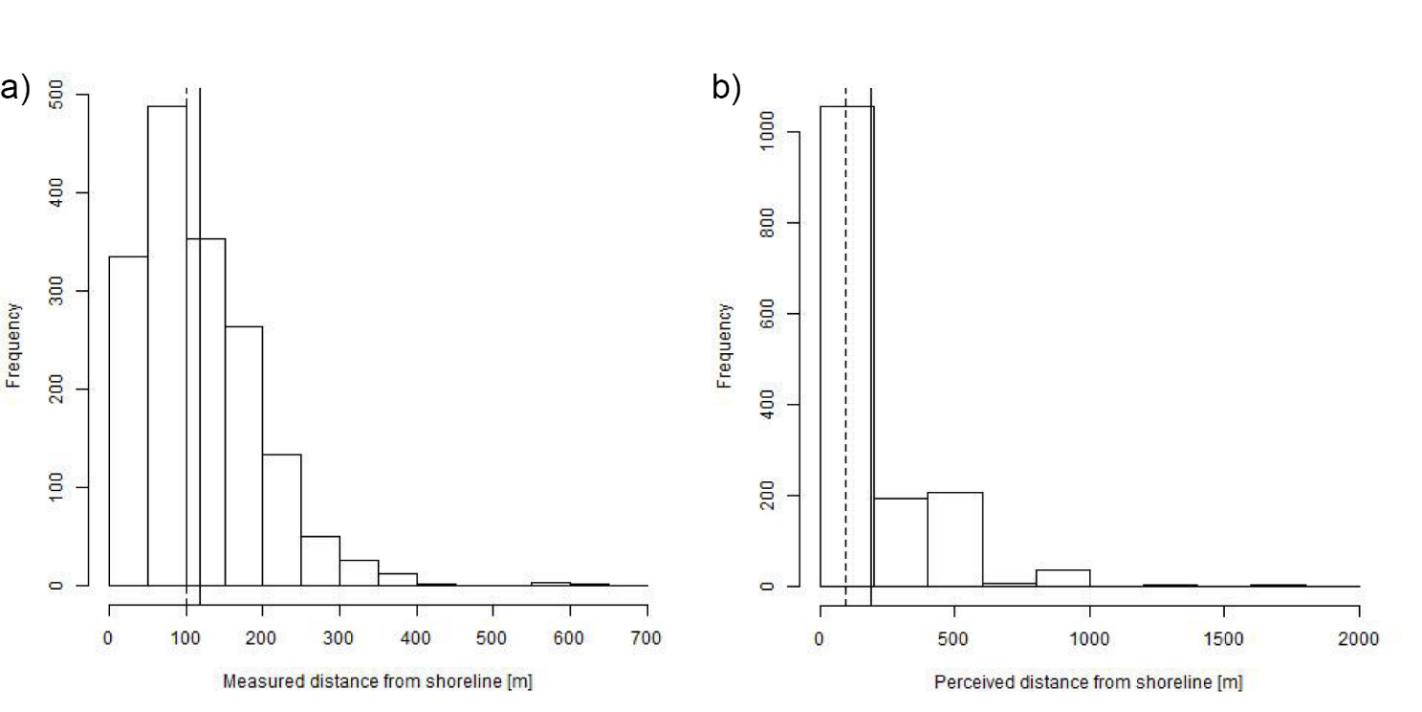


Fig. S 4: Measured distance of households from next closest riverbank of Jamuna River. Vertical lines: median (dashed) and mean (solid).

Table S 4: Overview of interview status of respondents in wave 2 – both for the overall sample and split by migration status.

Table S 5: Summary statistics of relevant variables.

| **Variable** | **N** | **Mean** | **Std. Dev.** | **Min** | **Pctl. 25** | **Pctl. 75** | **Max** |
| --- | --- | --- | --- | --- | --- | --- | --- |
| Any move between w1 and w2? | 1604 | 0.15 | 0.36 | 0 | 0 | 0 | 1 |
| Whole-household move between w1 and w2? | 1604 | 0.04 | 0.19 | 0 | 0 | 0 | 1 |
| Individual move between w1 and w2? | 1604 | 0.12 | 0.32 | 0 | 0 | 0 | 1 |
| Migration destination (rural or urban) | 1604 | 0.21 | 0.52 | 0 | 0 | 0 | 2 |
| Migration mode (whole-household or individual) | 1604 | 0.18 | 0.46 | 0 | 0 | 0 | 2 |
| Personal affectedness by erosion 2021 (w2) | 1604 | 0.33 | 0.47 | 0 | 0 | 1 | 1 |
| Severity of erosion affectedness (w2) | 1604 | 0.73 | 1.11 | 0 | 0 | 2 | 3 |
| Village-level share of erosion affectedness (w2) | 1604 | 0.33 | 0.26 | 0.02 | 0.12 | 0.48 | 1 |
| Personal affectedness by flood 2021 (w2) | 1603 | 0.48 | 0.5 | 0 | 0 | 1 | 1 |
| Severity of flood affectedness (w2) | 1597 | 0.82 | 0.97 | 0 | 0 | 2 | 3 |
| Village-level share of flood affectedness (w2) | 1604 | 0.48 | 0.19 | 0.12 | 0.32 | 0.61 | 0.88 |
| Migration aspirations (w1) | 1604 | 0.39 | 0.49 | 0 | 0 | 1 | 1 |
| Socio-economic status (w1) | 1369 | 0.04 | 1.32 | -3.28 | -0.82 | 0.68 | 9.46 |
| Sex | 1604 | 0.88 | 0.32 | 0 | 1 | 1 | 1 |
| Marital status | 1604 | 0.92 | 0.27 | 0 | 1 | 1 | 1 |
| Age | 1602 | 47.85 | 14.02 | 19 | 37.25 | 59 | 115 |
| Education level | 1603 | 0.87 | 1.34 | 0 | 0 | 1 | 5 |
| Income env.-dependent? (w1) | 1531 | 0.56 | 0.5 | 0 | 0 | 1 | 1 |
| Place attachment (w1) | 1603 | 4.4 | 0.73 | 1 | 4 | 5 | 5 |
| Risk preference (w1) | 1583 | 3.43 | 1.32 | 1 | 2 | 4 | 5 |
| Distance to river 2021 | 1585 | 119.19 | 80.25 | 1.01 | 60.72 | 163.07 | 689.42 |

Table S 6: Parameters assessed in the baseline questionnaire. Items in bold are re-assessed in the follow-up questionnaire.

| Section | Variables |
| --- | --- |
| 1 Demographics and socio-economic status | Age; Sex; Education; Religion; Faith in God; **Attachment to place; Life satisfaction**; **Happiness;** **Marital status** |
| 2 Household composition and migration history | **Household size;** **Migration of whole household; Migration of household members** |
| 3 Livelihood strategies, resources and assets | **Diversity of livelihood strategies; Financial resources; Assets**; Characteristics of land owned/used |
| 4 Migration aspirations | **Current aspirations, past aspirations** |
| 5 Perceptions | **Perceptions of different environmental** and climatic changes |
| 6 Riverbank erosion | **Perception; Affectedness; Adaptive strategies; Future risk** |
| 7 Risk preference | Choice in an investment game; Self-assessment |
| 8 Flooding | **Perception; Affectedness; Adaptive strategies; Future risk** |
| 9 Understanding of erosion and climate change | Causes of riverbank erosion and flooding; Familiarity with climate change |
| 0 Observed respondent characteristics | **Household economic status**; **Type of households in neighborhood; Access to electricity and safe drinking water in neighborhood** |

Table S 7: Impact types used to construct four categories of impact severity.

| **Impact severity** | **Impact type** |
| --- | --- |
| Strong impact | - Loss of house - Permanent displacement - Total loss of land |
| Medium impact | - Damage to house - Temporal displacement - Partial loss of land - Total loss of crop - Death/disease of animals - Loss of assets |
| Low impact | - Partial loss of crop - Decrease of soil quality - Difficulties with transport - Disease/injury of household members - Reduced income - Reduced food intake - Mental health impacts - Other impact/inconvenience |
| No impact | - No impact reported |

Fig. S 5: Most important impacts of the 2021 erosion and flood, respectively, as self-reported by respondents.

Table S 8: Dimensions and components of principal component analysis used to assess respondents’ socio-economic status.

| **Dimension** | **Components** |
| --- | --- |
| Valuables | 1.5*number of mobile phones + radio + 2*TV + 3*laptop + fan + battery + 2*solar panel + lighbulb + watch |
| Lifestock units (LSU) | 1*number of cows + 0.1*number of goats + 0.014* number of hens + 0.005*number of pigeons |
| Transport equipment | bicycle + 1.5*rickshaw + 1.5*van + 2*autobike + 2*motorbike + 3*CNG |
| Productive equipment | tractor + fishing net + boat |
| Total land size [ha] | - |
| Building quality | quality of roof (1=hay / tin; 1.5=wood / plastic; 2=concrete / steel; 3=tiles) +  quality of walls (1=jute / mud / bamboo; 1.5=wood; 2=concrete / iron; 3=brick) |
| Housing quality | house owned + 0.5*latrine + 0.5*connection to electricity grid |

Table S 9: Correlation matrix of main variables.

Appendix D: Supporting information for Results section

Table S 10: Influence of binary flood and erosion impact on whether a respondent migrated between wave 1 and wave 2 (linear regressions).

|  | | | | | | |
| --- | --- | --- | --- | --- | --- | --- |
|  | *Dependent variable:* | | | | | |
|  |  | | | | | |
|  | Migrated between w1 and w2? (n/y) | | | | | |
|  | (1) | (2) | (3) | (4) | (5) | (6) |
|  | | | | | | |
| Erosion impact (n/y) | 0.14^***^ | 0.13^***^ | 0.15^***^ | 0.14^***^ | 0.14^***^ | 0.14^***^ |
|  | (0.03) | (0.02) | (0.02) | (0.02) | (0.02) | (0.02) |
|  |  |  |  |  |  |  |
| Flood impact (n/y) | 0.02 | 0.02 | 0.02 | 0.02 | 0.02 | 0.02 |
|  | (0.03) | (0.03) | (0.02) | (0.02) | (0.03) | (0.02) |
|  |  |  |  |  |  |  |
| Aspirations (n/y) |  |  |  | 0.03 | 0.03 | 0.03 |
|  |  |  |  | (0.02) | (0.02) | (0.02) |
|  |  |  |  |  |  |  |
| Socio-econ. status |  |  |  | -0.02^**^ | -0.02^*^ | -0.01 |
|  |  |  |  | (0.01) | (0.01) | (0.01) |
|  |  |  |  |  |  |  |
| Sex (f/m) |  |  |  | 0.13^***^ | 0.14^***^ | 0.13^***^ |
|  |  |  |  | (0.04) | (0.04) | (0.04) |
|  |  |  |  |  |  |  |
| Married? (n/y) |  |  |  | -0.003 | -0.02 | 0.02 |
|  |  |  |  | (0.05) | (0.05) | (0.05) |
|  |  |  |  |  |  |  |
| Age: 31-40 yr |  |  |  | -0.03 | -0.05 | -0.03 |
|  |  |  |  | (0.04) | (0.04) | (0.04) |
|  |  |  |  |  |  |  |
| Age: 41-50 yr |  |  |  | -0.07^*^ | -0.12^***^ | -0.09^**^ |
|  |  |  |  | (0.04) | (0.04) | (0.04) |
|  |  |  |  |  |  |  |
| Age: 51-60 yr |  |  |  | -0.15^***^ | -0.21^***^ | -0.14^***^ |
|  |  |  |  | (0.04) | (0.04) | (0.04) |
|  |  |  |  |  |  |  |
| Age: 61+ yr |  |  |  | -0.18^***^ | -0.23^***^ | -0.19^***^ |
|  |  |  |  | (0.04) | (0.04) | (0.04) |
|  |  |  |  |  |  |  |
| Educ: primary |  |  |  | 0.02 | 0.01 | 0.02 |
|  |  |  |  | (0.03) | (0.03) | (0.03) |
|  |  |  |  |  |  |  |
| Educ: secondary |  |  |  | 0.03 | 0.04 | 0.03 |
|  |  |  |  | (0.03) | (0.04) | (0.04) |
|  |  |  |  |  |  |  |
| Educ: SSC passed |  |  |  | -0.05 | -0.12^*^ | -0.09 |
|  |  |  |  | (0.06) | (0.07) | (0.07) |
|  |  |  |  |  |  |  |
| Educ: HSC passed |  |  |  | 0.04 | 0.03 | 0.01 |
|  |  |  |  | (0.06) | (0.06) | (0.06) |
|  |  |  |  |  |  |  |
| Educ: university |  |  |  | 0.02 | -0.01 | 0.03 |
|  |  |  |  | (0.06) | (0.06) | (0.07) |
|  |  |  |  |  |  |  |
| Income env.-dep.? (n/y) |  |  |  | 0.04 | 0.04^*^ | 0.04 |
|  |  |  |  | (0.02) | (0.03) | (0.03) |
|  |  |  |  |  |  |  |
| Attachment (1-5) |  |  |  | -0.002 | 0.003 | -0.01 |
|  |  |  |  | (0.01) | (0.01) | (0.02) |
|  |  |  |  |  |  |  |
| Risk pref. (1-5) |  |  |  | 0.002 | -0.01 | -0.01 |
|  |  |  |  | (0.01) | (0.01) | (0.01) |
|  |  |  |  |  |  |  |
| Distance to river (m) |  |  |  | -0.0002^*^ | -0.0001 | -0.0003^**^ |
|  |  |  |  | (0.0001) | (0.0002) | (0.0001) |
|  |  |  |  |  |  |  |
| District: Gaibandha |  |  |  | 0.0001 | 0.02 | -0.03 |
|  |  |  |  | (0.06) | (0.07) | (0.07) |
|  |  |  |  |  |  |  |
| District: Jamalpur |  |  |  | 0.04 | 0.05 | 0.04 |
|  |  |  |  | (0.05) | (0.06) | (0.06) |
|  |  |  |  |  |  |  |
| District: Kurigram |  |  |  | 0.09^**^ | 0.11^***^ | 0.04 |
|  |  |  |  | (0.04) | (0.04) | (0.04) |
|  |  |  |  |  |  |  |
| District: Manikganj |  |  |  | 0.01 | 0.01 | -0.02 |
|  |  |  |  | (0.05) | (0.06) | (0.06) |
|  |  |  |  |  |  |  |
| District: Sirajganj |  |  |  | 0.01 | 0.04 | -0.03 |
|  |  |  |  | (0.05) | (0.05) | (0.05) |
|  |  |  |  |  |  |  |
| District: Tangail |  |  |  | 0.02 | 0.02 | 0.03 |
|  |  |  |  | (0.04) | (0.04) | (0.05) |
|  |  |  |  |  |  |  |
| Intercept | 0.10^***^ | 0.12^***^ | 0.11^***^ | 0.02 | 0.06 | 0.12 |
|  | (0.01) | (0.02) | (0.02) | (0.09) | (0.10) | (0.10) |
|  |  |  |  |  |  |  |
|  | | | | | | |
| Controls | No | No | No | Yes | Yes | Yes |
| Weights | No | Erosion | Flood | No | Erosion | Flood |
| Mean erosion control group | 0.11 | 0.11 | 0.11 | 0.11 | 0.11 | 0.11 |
| SD erosion control group | 0.31 | 0.32 | 0.32 | 0.31 | 0.32 | 0.32 |
| Observations | 1,595 | 1,271 | 1,271 | 1,271 | 1,271 | 1,271 |
| Log Likelihood | -606.98 | -848.47 | -701.56 | -478.66 | -795.97 | -668.70 |
| Akaike Inf. Crit. | 1,219.96 | 1,702.94 | 1,409.11 | 1,009.33 | 1,643.94 | 1,389.40 |
|  | | | | | | |
| *Note:* | ^*^p<0.1; ^**^p<0.05; ^***^p<0.01 | | | | | |
|  | Standard errors clustered by village. Baseline age: 18-30 yr. Baseline education: no education. Baseline district: Bogra. (n/y) – (no/yes), (f/m) – (female/male), (m) - (meters). | | | | | |

Table S 11: Influence of different flood and erosion impact categories on whether a respondent migrated between wave 1 and wave 2 (linear regressions).

|  | | | | | | |
| --- | --- | --- | --- | --- | --- | --- |
|  | *Dependent variable:* | | | | | |
|  |  | | | | | |
|  | Migrated between w1 and w2? (n/y) | | | | | |
|  | (1) | (2) | (3) | (4) | (5) | (6) |
|  | | | | | | |
| Erosion: some impact | 0.04 | 0.03 | 0.04 | 0.04 | 0.04 | 0.03 |
|  | (0.04) | (0.05) | (0.05) | (0.05) | (0.05) | (0.05) |
|  |  |  |  |  |  |  |
| Erosion: medium impact | 0.09^***^ | 0.09^***^ | 0.09^***^ | 0.10^***^ | 0.09^***^ | 0.08^***^ |
|  | (0.03) | (0.03) | (0.03) | (0.03) | (0.03) | (0.03) |
|  |  |  |  |  |  |  |
| Erosion: strong impact | 0.19^***^ | 0.18^***^ | 0.21^***^ | 0.21^***^ | 0.21^***^ | 0.22^***^ |
|  | (0.05) | (0.03) | (0.03) | (0.04) | (0.03) | (0.03) |
|  |  |  |  |  |  |  |
| Flood: some impact | 0.004 | -0.003 | -0.003 | 0.01 | -0.003 | -0.002 |
|  | (0.03) | (0.03) | (0.03) | (0.03) | (0.03) | (0.03) |
|  |  |  |  |  |  |  |
| Flood: medium impact | 0.04 | 0.04 | 0.03 | 0.03 | 0.03 | 0.02 |
|  | (0.04) | (0.03) | (0.03) | (0.03) | (0.03) | (0.03) |
|  |  |  |  |  |  |  |
| Flood: strong impact | 0.06 | 0.07 | 0.06 | 0.11^**^ | 0.11^***^ | 0.08^*^ |
|  | (0.05) | (0.04) | (0.05) | (0.05) | (0.04) | (0.05) |
|  |  |  |  |  |  |  |
| Aspirations (n/y) |  |  |  | 0.02 | 0.02 | 0.02 |
|  |  |  |  | (0.02) | (0.02) | (0.02) |
|  |  |  |  |  |  |  |
| Socio-econ. status |  |  |  | -0.02^**^ | -0.01 | -0.01 |
|  |  |  |  | (0.01) | (0.01) | (0.01) |
|  |  |  |  |  |  |  |
| Sex (f/m) |  |  |  | 0.13^***^ | 0.14^***^ | 0.12^***^ |
|  |  |  |  | (0.04) | (0.04) | (0.04) |
|  |  |  |  |  |  |  |
| Married? (n/y) |  |  |  | -0.004 | -0.02 | 0.02 |
|  |  |  |  | (0.05) | (0.05) | (0.05) |
|  |  |  |  |  |  |  |
| Age: 31-40 yr |  |  |  | -0.03 | -0.05 | -0.03 |
|  |  |  |  | (0.04) | (0.04) | (0.04) |
|  |  |  |  |  |  |  |
| Age: 41-50 yr |  |  |  | -0.07^*^ | -0.12^***^ | -0.09^**^ |
|  |  |  |  | (0.04) | (0.04) | (0.04) |
|  |  |  |  |  |  |  |
| Age: 51-60 yr |  |  |  | -0.15^***^ | -0.21^***^ | -0.14^***^ |
|  |  |  |  | (0.04) | (0.04) | (0.04) |
|  |  |  |  |  |  |  |
| Age: 61+ yr |  |  |  | -0.18^***^ | -0.24^***^ | -0.20^***^ |
|  |  |  |  | (0.04) | (0.04) | (0.04) |
|  |  |  |  |  |  |  |
| Educ: primary |  |  |  | 0.01 | 0.01 | 0.01 |
|  |  |  |  | (0.03) | (0.03) | (0.03) |
|  |  |  |  |  |  |  |
| Educ: secondary |  |  |  | 0.03 | 0.03 | 0.03 |
|  |  |  |  | (0.03) | (0.04) | (0.04) |
|  |  |  |  |  |  |  |
| Educ: SSC passed |  |  |  | -0.04 | -0.11 | -0.08 |
|  |  |  |  | (0.06) | (0.07) | (0.07) |
|  |  |  |  |  |  |  |
| Educ: HSC passed |  |  |  | 0.04 | 0.03 | 0.01 |
|  |  |  |  | (0.06) | (0.06) | (0.06) |
|  |  |  |  |  |  |  |
| Educ: university |  |  |  | 0.02 | -0.01 | 0.03 |
|  |  |  |  | (0.06) | (0.06) | (0.07) |
|  |  |  |  |  |  |  |
| Income env.-dep.? (n/y) |  |  |  | 0.04^*^ | 0.05^*^ | 0.04 |
|  |  |  |  | (0.02) | (0.03) | (0.03) |
|  |  |  |  |  |  |  |
| Attachment (1-5) |  |  |  | -0.005 | 0.001 | -0.01 |
|  |  |  |  | (0.01) | (0.01) | (0.02) |
|  |  |  |  |  |  |  |
| Risk pref. (1-5) |  |  |  | -0.0001 | -0.01 | -0.01 |
|  |  |  |  | (0.01) | (0.01) | (0.01) |
|  |  |  |  |  |  |  |
| Distance to river (m) |  |  |  | -0.0002^*^ | -0.0001 | -0.0003^**^ |
|  |  |  |  | (0.0001) | (0.0002) | (0.0001) |
|  |  |  |  |  |  |  |
| District: Gaibandha |  |  |  | 0.01 | 0.04 | -0.02 |
|  |  |  |  | (0.06) | (0.07) | (0.07) |
|  |  |  |  |  |  |  |
| District: Jamalpur |  |  |  | 0.04 | 0.06 | 0.05 |
|  |  |  |  | (0.05) | (0.06) | (0.06) |
|  |  |  |  |  |  |  |
| District: Kurigram |  |  |  | 0.09^**^ | 0.12^***^ | 0.05 |
|  |  |  |  | (0.04) | (0.04) | (0.04) |
|  |  |  |  |  |  |  |
| District: Manikganj |  |  |  | 0.02 | 0.02 | 0.001 |
|  |  |  |  | (0.05) | (0.06) | (0.06) |
|  |  |  |  |  |  |  |
| District: Sirajganj |  |  |  | 0.01 | 0.04 | -0.04 |
|  |  |  |  | (0.05) | (0.05) | (0.05) |
|  |  |  |  |  |  |  |
| District: Tangail |  |  |  | 0.004 | -0.004 | -0.001 |
|  |  |  |  | (0.04) | (0.05) | (0.05) |
|  |  |  |  |  |  |  |
| Intercept | 0.10^***^ | 0.13^***^ | 0.12^***^ | 0.04 | 0.08 | 0.15 |
|  | (0.01) | (0.02) | (0.02) | (0.09) | (0.10) | (0.10) |
|  |  |  |  |  |  |  |
|  | | | | | | |
| Controls | No | No | No | Yes | Yes | Yes |
| Weights | No | Erosion | Flood | No | Erosion | Flood |
| Mean erosion control group | 0.11 | 0.11 | 0.11 | 0.11 | 0.11 | 0.11 |
| SD erosion control group | 0.31 | 0.32 | 0.32 | 0.31 | 0.32 | 0.32 |
| Observations | 1,589 | 1,265 | 1,265 | 1,265 | 1,265 | 1,265 |
| Log Likelihood | -596.60 | -837.51 | -691.24 | -467.69 | -778.80 | -655.67 |
| Akaike Inf. Crit. | 1,207.20 | 1,689.02 | 1,396.48 | 995.38 | 1,617.59 | 1,371.34 |
|  | | | | | | |
| *Note:* | ^*^p<0.1; ^**^p<0.05; ^***^p<0.01 | | | | | |
|  | Standard errors clustered by village. Baseline erosion/flood impact: no impact. Baseline age: 18-30 yr. Baseline education: no education. Baseline district: Bogra. (n/y) – (no/yes), (f/m) – (female/male), (m) - (meters). | | | | | |

Table S 12: Influence of binary flood and erosion impact on migration destination (multinomial logit models).

|  | | | | | | |
| --- | --- | --- | --- | --- | --- | --- |
|  | *Dependent variable:* | | | | | |
|  |  | | | | | |
|  | Migration destination | | | | | |
|  | (1) | (2) | (3) | (4) | (5) | (6) |
|  | | | | | | |
| Erosion impact-rural | 1.22^***^ | 1.14^***^ | 1.19^***^ | 1.28^***^ | 1.29^***^ | 1.14^***^ |
|  | (0.19) | (0.20) | (0.20) | (0.21) | (0.21) | (0.21) |
|  |  |  |  |  |  |  |
| Flood impact-rural | 0.30 | 0.32 | 0.29 | 0.35 | 0.36^*^ | 0.31 |
|  | (0.19) | (0.20) | (0.20) | (0.21) | (0.21) | (0.21) |
|  |  |  |  |  |  |  |
| Aspirations-rural |  |  |  | 0.07 | 0.18 | 0.10 |
|  |  |  |  | (0.20) | (0.20) | (0.20) |
|  |  |  |  |  |  |  |
| Socio-econ. status-rural |  |  |  | -0.10 | -0.03 | 0.02 |
|  |  |  |  | (0.08) | (0.08) | (0.08) |
|  |  |  |  |  |  |  |
| Erosion impact-urban | 0.52^**^ | 0.25 | 0.63^**^ | 0.54^*^ | 0.38 | 0.65^**^ |
|  | (0.25) | (0.26) | (0.27) | (0.30) | (0.29) | (0.29) |
|  |  |  |  |  |  |  |
| Flood impact-urban | -0.03 | -0.17 | -0.26 | -0.10 | -0.15 | -0.34 |
|  | (0.24) | (0.25) | (0.27) | (0.28) | (0.27) | (0.28) |
|  |  |  |  |  |  |  |
| Aspirations-urban |  |  |  | 0.40 | 0.28 | 0.47^*^ |
|  |  |  |  | (0.26) | (0.25) | (0.25) |
|  |  |  |  |  |  |  |
| Socio-econ. status-urban |  |  |  | -0.29^**^ | -0.27^**^ | -0.28^**^ |
|  |  |  |  | (0.12) | (0.12) | (0.12) |
|  |  |  |  |  |  |  |
|  | | | | | | |
| Controls | No | No | No | Yes | Yes | Yes |
| Weights | No | Erosion | Flood | No | Erosion | Flood |
| Observations | 1,595 | 1,271 | 1,271 | 1,271 | 1,271 | 1,271 |
| R^2^ | 0.04 | -0.10 | -0.07 | 0.13 | 0.01 | 0.004 |
| Log Likelihood | -808.27 | -771.59 | -752.31 | -612.46 | -691.75 | -698.94 |
| LR Test | 66.71^***^ (df = 6) | -139.31 (df = 6) | -100.73 (df = 6) | 178.96^***^ (df = 52) | 20.37 (df = 52) | 6.01 (df = 52) |
|  | | | | | | |
| *Note:* | ^*^p<0.1; ^**^p<0.05; ^***^p<0.01 | | | | | |
|  | Baseline DV: no move. (n/y) – (no/yes). | | | | | |

Table S 13: Influence of binary flood and erosion impact on migration mode (multinomial logit models).

|  | | | | | | |
| --- | --- | --- | --- | --- | --- | --- |
|  | *Dependent variable:* | | | | | |
|  |  | | | | | |
|  | Migration mode | | | | | |
|  | (1) | (2) | (3) | (4) | (5) | (6) |
|  | | | | | | |
| Erosion impact-individual | 0.57^***^ | 0.43^**^ | 0.58^***^ | 0.66^***^ | 0.64^***^ | 0.75^***^ |
|  | (0.17) | (0.18) | (0.18) | (0.21) | (0.20) | (0.20) |
|  |  |  |  |  |  |  |
| Flood impact-individual | 0.33^*^ | 0.31^*^ | 0.24 | 0.29 | 0.27 | 0.23 |
|  | (0.17) | (0.18) | (0.18) | (0.20) | (0.20) | (0.20) |
|  |  |  |  |  |  |  |
| Aspirations-individual |  |  |  | 0.01 | -0.06 | 0.03 |
|  |  |  |  | (0.19) | (0.19) | (0.19) |
|  |  |  |  |  |  |  |
| Socio-econ. status-individual |  |  |  | -0.19^**^ | -0.16^**^ | -0.20^**^ |
|  |  |  |  | (0.08) | (0.08) | (0.08) |
|  |  |  |  |  |  |  |
| Erosion impact-whole-household | 3.06^***^ | 3.36^***^ | 3.09^***^ | 2.73^***^ | 3.10^***^ | 2.70^***^ |
|  | (0.46) | (0.56) | (0.51) | (0.51) | (0.59) | (0.52) |
|  |  |  |  |  |  |  |
| Flood impact-whole-household | -0.50 | -0.42 | -0.38 | -0.40 | -0.37 | -0.47 |
|  | (0.32) | (0.34) | (0.35) | (0.36) | (0.37) | (0.37) |
|  |  |  |  |  |  |  |
| Aspirations-whole-household |  |  |  | 0.58^*^ | 0.55 | 0.44 |
|  |  |  |  | (0.34) | (0.35) | (0.35) |
|  |  |  |  |  |  |  |
| Socio-econ. status-whole-household |  |  |  | -0.02 | -0.04 | 0.14 |
|  |  |  |  | (0.14) | (0.14) | (0.13) |
|  |  |  |  |  |  |  |
|  | | | | | | |
| Controls | No | No | No | Yes | Yes | Yes |
| Weights | No | Erosion | Flood | No | Erosion | Flood |
| Observations | 1,595 | 1,271 | 1,271 | 1,271 | 1,271 | 1,271 |
| R^2^ | 0.06 | -0.09 | -0.08 | 0.19 | 0.08 | 0.07 |
| Log Likelihood | -749.04 | -727.07 | -719.11 | -541.92 | -612.94 | -619.24 |
| LR Test | 91.97^***^ (df = 6) | -121.65 (df = 6) | -105.73 (df = 6) | 248.65^***^ (df = 52) | 106.61^***^ (df = 50) | 94.01^***^ (df = 50) |
|  | | | | | | |
| *Note:* | ^*^p<0.1; ^**^p<0.05; ^***^p<0.01 | | | | | |
|  | Baseline DV: no move. (n/y) – (no/yes). Models 5 and 6 do not include the covariate 'distance to riverbank' due to convergence issues. | | | | | |


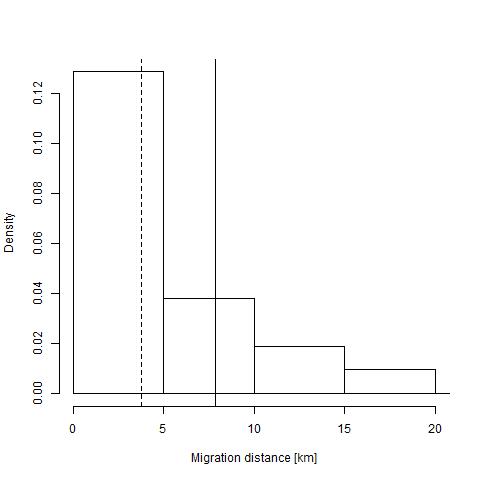


Fig. S 6: Histogram of migration distance of whole-household migrants. Distances > 20 km are excluded from the visual display to enhance readability, resulting in the exclusion of 2.4% of observations. Vertical lines: median (dashed) and mean (solid).

Appendix E: Robustness checks

The robustness section provides evidence on four aspects: a) logistic instead of linear regressions; b) considering village-level affectedness; c) different specifications of the dependent variable; and d) different specifications of the independent variables. Overall, these tests support my main finding of a strong link between erosion exposure and migration, and a weak or non-existent relationship between flood exposure and migration.

E.1 Logistic regressions

First, logistic regression models might be more appropriate for binary outcome variables such as “migrated: yes or no”. Table S 14 and Table S 15 contain logit models of the influence of erosion/flood impact on the migration likelihood. Results are substantively and statistically comparable to Table S 10 and Table S 11, indicating that the modeling choice does not affect the conclusions.

Table S 14: Influence of binary flood and erosion impact on whether a respondent migrated between wave 1 and wave 2 (logistic regressions).

|  | | | | | | |
| --- | --- | --- | --- | --- | --- | --- |
|  | *Dependent variable:* | | | | | |
|  |  | | | | | |
|  | Migrated between w1 and w2? (n/y) | | | | | |
|  | (1) | (2) | (3) | (4) | (5) | (6) |
|  | | | | | | |
| Erosion impact (n/y) | 0.98^***^ | 0.84^***^ | 1.00^***^ | 1.03^***^ | 0.98^***^ | 0.97^***^ |
|  | (0.22) | (0.18) | (0.15) | (0.18) | (0.19) | (0.17) |
|  |  |  |  |  |  |  |
| Flood impact (n/y) | 0.18 | 0.15 | 0.11 | 0.18 | 0.16 | 0.10 |
|  | (0.23) | (0.21) | (0.15) | (0.18) | (0.22) | (0.15) |
|  |  |  |  |  |  |  |
| Aspirations (n/y) |  |  |  | 0.18 | 0.20 | 0.21 |
|  |  |  |  | (0.17) | (0.19) | (0.16) |
|  |  |  |  |  |  |  |
| Socio-econ. status |  |  |  | -0.16^**^ | -0.11 | -0.06 |
|  |  |  |  | (0.07) | (0.08) | (0.07) |
|  |  |  |  |  |  |  |
|  | | | | | | |
| Controls | No | No | No | Yes | Yes | Yes |
| Weights | No | Erosion | Flood | No | Erosion | Flood |
| Observations | 1,595 | 1,271 | 1,271 | 1,271 | 1,271 | 1,271 |
| Log Likelihood | -656.01 | -379.77 | -608.27 | -503.30 | -345.54 | -575.52 |
| Akaike Inf. Crit. | 1,318.02 | 765.55 | 1,222.53 | 1,058.61 | 743.09 | 1,203.03 |
|  | | | | | | |
| *Note:* | ^*^p<0.1; ^**^p<0.05; ^***^p<0.01 | | | | | |
|  | Standard errors clustered by village. (n/y) – (no/yes). | | | | | |

Table S 15: Influence of the severity of flood and erosion impacts on whether a respondent migrated between wave 1 and wave 2 (logistic regressions).

|  | | | | | | |
| --- | --- | --- | --- | --- | --- | --- |
|  | *Dependent variable:* | | | | | |
|  |  | | | | | |
|  | Migrated between w1 and w2? (n/y) | | | | | |
|  | (1) | (2) | (3) | (4) | (5) | (6) |
|  | | | | | | |
| Erosion: some impact | 0.40 | 0.26 | 0.34 | 0.40 | 0.34 | 0.33 |
|  | (0.28) | (0.40) | (0.34) | (0.41) | (0.42) | (0.35) |
|  |  |  |  |  |  |  |
| Erosion: medium impact | 0.70^***^ | 0.58^***^ | 0.65^***^ | 0.75^***^ | 0.64^***^ | 0.59^***^ |
|  | (0.24) | (0.21) | (0.18) | (0.22) | (0.23) | (0.19) |
|  |  |  |  |  |  |  |
| Erosion: strong impact | 1.24^***^ | 1.04^***^ | 1.26^***^ | 1.43^***^ | 1.43^***^ | 1.40^***^ |
|  | (0.29) | (0.23) | (0.19) | (0.26) | (0.27) | (0.22) |
|  |  |  |  |  |  |  |
| Flood: some impact | 0.05 | -0.03 | -0.03 | 0.06 | -0.04 | -0.03 |
|  | (0.26) | (0.25) | (0.20) | (0.23) | (0.27) | (0.21) |
|  |  |  |  |  |  |  |
| Flood: medium impact | 0.31 | 0.22 | 0.18 | 0.24 | 0.21 | 0.15 |
|  | (0.27) | (0.23) | (0.18) | (0.21) | (0.25) | (0.19) |
|  |  |  |  |  |  |  |
| Flood: strong impact | 0.40 | 0.42 | 0.35 | 0.74^**^ | 0.76^**^ | 0.53^*^ |
|  | (0.32) | (0.33) | (0.30) | (0.34) | (0.36) | (0.32) |
|  |  |  |  |  |  |  |
| Aspirations (n/y) |  |  |  | 0.13 | 0.12 | 0.14 |
|  |  |  |  | (0.17) | (0.19) | (0.16) |
|  |  |  |  |  |  |  |
| Socio-econ. status |  |  |  | -0.13^*^ | -0.07 | -0.03 |
|  |  |  |  | (0.07) | (0.08) | (0.07) |
|  |  |  |  |  |  |  |
|  | | | | | | |
| Controls | No | No | No | Yes | Yes | Yes |
| Weights | No | Erosion | Flood | No | Erosion | Flood |
| Observations | 1,589 | 1,265 | 1,265 | 1,265 | 1,265 | 1,265 |
| Log Likelihood | -648.68 | -373.23 | -600.96 | -494.32 | -333.83 | -564.82 |
| Akaike Inf. Crit. | 1,311.36 | 760.47 | 1,215.92 | 1,048.64 | 727.65 | 1,189.64 |
|  | | | | | | |
| *Note:* | ^*^p<0.1; ^**^p<0.05; ^***^p<0.01 | | | | | |
|  | Standard errors clustered by village. Baseline erosion/flood impact: no impact. (n/y) – (no/yes). | | | | | |

E.2 Village-level affectedness

Second, floods and erosion materialize at the village level, affecting certain villages more strongly than others. It might, hence, be that severe flood/erosion impacts in the village make people move even if their household has not been affected directly. Such an effect might occur if people extrapolate erosion/flood occurrence in the previous monsoon to a potential risk of being affected in the upcoming monsoon season, and hence move to prevent being affected. Table S 16 presents models equivalent to those in Table S 10, but including controls for village-level affectedness by erosion/floods (i.e., the percentage of respondents from the respective village who indicated that they had been affected by floods/erosion). Indeed, village-level erosion affectedness has a significant and substantive effect on the migration likelihood, albeit not consistently across model specifications. This indicates that even if a household has not been affected itself, the likelihood to migrate increases if a larger share of the village has been affected by erosion. Nevertheless, the effect of household-level erosion affectedness remains significant throughout the model specifications. Overall, it appears that there are two pathways by which erosion affects migration: directly through household-level affectedness, and indirectly through village-level impacts. For flooding, neither the village-level nor direct affectedness exhibit a significant influence on migration behavior. These results are supported by the findings of Gray and Mueller (2012) who show that subdistrict exposure to crop failure can have a stronger influence on population mobility in Bangladesh than actual household exposure.

Table S 16: Influence of binary flood and erosion impact on whether a respondent migrated or shifted their house between wave 1 and wave 2, controlling for village-level affectedness by floods/erosion (linear regressions).

|  | | | | | | |
| --- | --- | --- | --- | --- | --- | --- |
|  | *Dependent variable:* | | | | | |
|  |  | | | | | |
|  | Migrated between w1 and w2? (n/y) | | | | | |
|  | (1) | (2) | (3) | (4) | (5) | (6) |
|  | | | | | | |
| Erosion impact (n/y) | 0.13^***^ | 0.11^***^ | 0.12^***^ | 0.13^***^ | 0.12^***^ | 0.11^***^ |
|  | (0.03) | (0.03) | (0.03) | (0.03) | (0.03) | (0.03) |
|  |  |  |  |  |  |  |
| Flood impact (n/y) | 0.02 | 0.02 | 0.02 | 0.02 | 0.02 | 0.02 |
|  | (0.03) | (0.03) | (0.02) | (0.02) | (0.03) | (0.02) |
|  |  |  |  |  |  |  |
| Erosion: Share affected HH in village (%) | 0.02 | 0.07 | 0.10^*^ | 0.05 | 0.09 | 0.14^**^ |
|  | (0.08) | (0.06) | (0.06) | (0.06) | (0.06) | (0.06) |
|  |  |  |  |  |  |  |
| Flood: Share affected HH in village (%) | 0.03 | -0.04 | -0.06 | 0.003 | 0.004 | -0.09 |
|  | (0.09) | (0.09) | (0.08) | (0.09) | (0.10) | (0.08) |
|  |  |  |  |  |  |  |
| Aspirations (n/y) |  |  |  | 0.03 | 0.03 | 0.02 |
|  |  |  |  | (0.02) | (0.02) | (0.02) |
|  |  |  |  |  |  |  |
| Socio-econ. status |  |  |  | -0.02^**^ | -0.01^*^ | -0.01 |
|  |  |  |  | (0.01) | (0.01) | (0.01) |
|  |  |  |  |  |  |  |
|  | | | | | | |
| Controls | No | No | No | Yes | Yes | Yes |
| Weights | No | Erosion | Flood | No | Erosion | Flood |
| Mean erosion control group | 0.11 | 0.11 | 0.11 | 0.11 | 0.11 | 0.11 |
| SD erosion control group | 0.31 | 0.32 | 0.32 | 0.31 | 0.32 | 0.32 |
| Observations | 1,595 | 1,271 | 1,271 | 1,271 | 1,271 | 1,271 |
| Log Likelihood | -606.40 | -847.68 | -699.87 | -478.05 | -794.13 | -666.09 |
| Akaike Inf. Crit. | 1,222.80 | 1,705.37 | 1,409.74 | 1,012.10 | 1,644.26 | 1,388.17 |
|  | | | | | | |
| *Note:* | ^*^p<0.1; ^**^p<0.05; ^***^p<0.01 | | | | | |
|  | Standard errors clustered by village. (n/y) – (no/yes). | | | | | |

E.3 Different specifications of migration (dependent variable)

Third, I investigate different specifications of the dependent variable. The results in Table S 10 and Table S 11 combine different forms of mobility: a) moving the whole household to a location outside of the village and b) keeping the household in the village while the household head moves individually to live somewhere else. Since these are very different forms of mobility, also the underlying relationships to environmental changes might differ. To test the influence of the specification of the outcome variable, I model the two mobility forms outlined above separately, and compare them to the estimates of the combined mobility variable. Erosion impact has a consistently positive impact on the likelihood to move (Table S 17). Substantively, however, the impact varies drastically between mobility forms: Moving the household outside the village gets nine times more likely if respondents have been affected by erosion, compared to the unaffected control group. For an individual move, the increase is only around 50%. The effects of flooding are inconsistent: They appear to decrease the probability to move with the whole household, while they increase the likelihood of an individual move. Prior aspirations slightly increase the likelihood to move with the whole household. Low socio-economic status does not exert a significant influence. Splitting the erosion/flood impacts by degree of severity reveals that medium and strong erosion impacts have a positive influence on both mobility forms (Table S 18). Medium and strong flood impacts make individual migration more likely, while medium and some flood impacts decrease the likelihood to migrate with the whole household outside of the village.

Next, the analyses have so far only considered individual migration of the responding household head. In many households, however, the main respondent has not migrated in the past year, while one or several family members (typically, a son) have temporarily moved elsewhere. Accordingly, I widen the definition of migration to “either the respondent or a household member migrated in the past year”. Results show that the effect of erosion affectedness gets relatively dampened compared to the narrower definition of only household head migration, but remains statistically significant (Table S 19). Likewise, flood affectedness has a consistently significant effect, albeit substantively weaker than that of erosion. Lastly, households whose income depends on the environment are significantly more likely to engage in migration. Note that these analyses only consider covariates which are measured at the household level, since the dependent variable considers household level migration.

Lastly, I widen the definition of migration to also include another form of mobility commonly observed along the Jamuna River. In light of the ongoing erosion, many houses are constructed in a way that they can easily be dismantled, moved away from the river and rebuilt. Such “shifting” of the household location has so far not been considered in the analyses since these households do not leave the administrative boundaries of the village and do hence not “migrate”. Nevertheless, shifting is an important adaptive strategy with respect to erosion and is hence included as a robustness check in Table S 20. The results are similar to Table S 10, but the effects are substantively more pronounced, especially for erosion affectedness: Having been affected by erosion more than triples the likelihood to migrate or to shift the house within the village.

Table S 17: Influence of binary flood and erosion impact on whether a respondent migrated outside the village between wave 1 and wave 2 (model 1), whether the whole household moved to a location outside the village (model 2) or whether the household stayed in the village while the respondent moved away individually (model 3). All models are linear regressions.

|  | | | |
| --- | --- | --- | --- |
|  | *Dependent variable:* | | |
|  |  | | |
|  | Any move (n/y) | Whole-household move (n/y) | Individual move (n/y) |
|  | (1) | (2) | (3) |
|  | | | |
| Erosion impact (n/y) | 0.14^***^ | 0.09^***^ | 0.05^***^ |
|  | (0.03) | (0.01) | (0.02) |
|  |  |  |  |
| Flood impact (n/y) | 0.02 | -0.02^*^ | 0.05^**^ |
|  | (0.04) | (0.01) | (0.02) |
|  |  |  |  |
| Aspirations (n/y) | 0.03 | 0.03^**^ | -0.0002 |
|  | (0.03) | (0.01) | (0.02) |
|  |  |  |  |
| Socio-econ. status | -0.02 | -0.004 | -0.01 |
|  | (0.01) | (0.01) | (0.01) |
|  |  |  |  |
|  | | | |
| Controls | Yes | Yes | Yes |
| Weights | Erosion | Erosion | Erosion |
| Mean erosion control group | 0.11 | 0.01 | 0.10 |
| SD erosion control group | 0.32 | 0.10 | 0.31 |
| Observations | 1,271 | 1,271 | 1,271 |
| Log Likelihood | -795.97 | -91.60 | -619.54 |
| Akaike Inf. Crit. | 1,643.94 | 235.20 | 1,291.07 |
|  | | | |
| *Note:* | ^*^p<0.1; ^**^p<0.05; ^***^p<0.01 | | |
|  | Standard errors clustered by village. (n/y) – (no/yes). | | |

Table S 18: Influence of the severity of flood and erosion impacts on whether a respondent migrated outside the village between wave 1 and wave 2 (model 1), whether the whole household moved to a location outside the village (model 2) or whether the household stayed in the village while the respondent moved away individually (model 3). All models are linear regressions.

|  | | | |
| --- | --- | --- | --- |
|  | *Dependent variable:* | | |
|  |  | | |
|  | Any move (n/y) | Whole-household move (n/y) | Individual move (n/y) |
|  | (1) | (2) | (3) |
|  | | | |
| Erosion: some impact | 0.04 | 0.06^**^ | -0.02 |
|  | (0.04) | (0.03) | (0.04) |
|  |  |  |  |
| Erosion: medium impact | 0.09^***^ | 0.03^**^ | 0.06^**^ |
|  | (0.04) | (0.01) | (0.02) |
|  |  |  |  |
| Erosion: strong impact | 0.21^***^ | 0.18^***^ | 0.04 |
|  | (0.04) | (0.02) | (0.03) |
|  |  |  |  |
| Flood: some impact | -0.003 | -0.03^**^ | 0.03 |
|  | (0.04) | (0.02) | (0.03) |
|  |  |  |  |
| Flood: medium impact | 0.03 | -0.03^**^ | 0.06^**^ |
|  | (0.05) | (0.02) | (0.03) |
|  |  |  |  |
| Flood: strong impact | 0.11^*^ | 0.04 | 0.08^**^ |
|  | (0.06) | (0.02) | (0.04) |
|  |  |  |  |
| Aspirations (n/y) | 0.02 | 0.03^**^ | -0.003 |
|  | (0.03) | (0.01) | (0.02) |
|  |  |  |  |
| Socio-econ. status | -0.01 | 0.0002 | -0.01 |
|  | (0.01) | (0.005) | (0.01) |
|  |  |  |  |
|  | | | |
| Controls | Yes | Yes | Yes |
| Weights | Erosion | Erosion | Erosion |
| Mean erosion control group | 0.11 | 0.01 | 0.10 |
| SD erosion control group | 0.32 | 0.10 | 0.31 |
| Observations | 1,265 | 1,265 | 1,265 |
| Log Likelihood | -778.80 | -33.35 | -616.49 |
| Akaike Inf. Crit. | 1,617.59 | 126.71 | 1,292.98 |
|  | | | |
| *Note:* | ^*^p<0.1; ^**^p<0.05; ^***^p<0.01 | | |
|  | Standard errors clustered by village. (n/y) – (no/yes). | | |

Table S 19: Influence of binary flood and erosion impact on whether a respondent or a household member migrated between wave 1 and wave 2 (linear regressions).

|  | | | | | | |
| --- | --- | --- | --- | --- | --- | --- |
|  | *Dependent variable:* | | | | | |
|  |  | | | | | |
|  | Someone from household migrated between w1 and w2? (n/y) | | | | | |
|  | (1) | (2) | (3) | (4) | (5) | (6) |
|  | | | | | | |
| Erosion impact (n/y) | 0.09^**^ | 0.08^***^ | 0.11^***^ | 0.11^***^ | 0.10^***^ | 0.11^***^ |
|  | (0.04) | (0.03) | (0.03) | (0.03) | (0.03) | (0.03) |
|  |  |  |  |  |  |  |
| Flood impact (n/y) | 0.06^*^ | 0.07^**^ | 0.06^**^ | 0.07^***^ | 0.07^**^ | 0.06^**^ |
|  | (0.03) | (0.03) | (0.03) | (0.03) | (0.03) | (0.03) |
|  |  |  |  |  |  |  |
| Socio-econ. status |  |  |  | -0.01 | -0.003 | 0.0001 |
|  |  |  |  | (0.01) | (0.01) | (0.01) |
|  |  |  |  |  |  |  |
| Income env.-dep.? (n/y) |  |  |  | 0.06^**^ | 0.08^***^ | 0.07^**^ |
|  |  |  |  | (0.03) | (0.03) | (0.03) |
|  |  |  |  |  |  |  |
| Distance to river (m) |  |  |  | -0.0002 | -0.0002 | -0.0003^**^ |
|  |  |  |  | (0.0002) | (0.0002) | (0.0002) |
|  |  |  |  |  |  |  |
| District: Gaibandha |  |  |  | 0.04 | -0.03 | -0.04 |
|  |  |  |  | (0.07) | (0.09) | (0.09) |
|  |  |  |  |  |  |  |
| District: Jamalpur |  |  |  | 0.11^*^ | 0.08 | 0.10 |
|  |  |  |  | (0.07) | (0.07) | (0.07) |
|  |  |  |  |  |  |  |
| District: Kurigram |  |  |  | 0.11^**^ | 0.09^*^ | 0.04 |
|  |  |  |  | (0.05) | (0.05) | (0.05) |
|  |  |  |  |  |  |  |
| District: Manikganj |  |  |  | -0.002 | -0.05 | -0.05 |
|  |  |  |  | (0.06) | (0.07) | (0.07) |
|  |  |  |  |  |  |  |
| District: Sirajganj |  |  |  | -0.02 | -0.02 | -0.05 |
|  |  |  |  | (0.06) | (0.06) | (0.06) |
|  |  |  |  |  |  |  |
| District: Tangail |  |  |  | -0.04 | -0.07 | -0.06 |
|  |  |  |  | (0.05) | (0.05) | (0.05) |
|  |  |  |  |  |  |  |
| Intercept | 0.23^***^ | 0.24^***^ | 0.23^***^ | 0.17^***^ | 0.18^***^ | 0.23^***^ |
|  | (0.02) | (0.03) | (0.02) | (0.05) | (0.06) | (0.06) |
|  |  |  |  |  |  |  |
|  | | | | | | |
| Controls | No | No | No | Yes | Yes | Yes |
| Weights | No | Erosion | Flood | No | Erosion | Flood |
| Mean erosion control group | 0.25 | 0.25 | 0.25 | 0.25 | 0.25 | 0.25 |
| SD erosion control group | 0.43 | 0.43 | 0.43 | 0.43 | 0.43 | 0.43 |
| Observations | 1,595 | 1,271 | 1,271 | 1,286 | 1,271 | 1,271 |
| Log Likelihood | -976.42 | -1,062.31 | -918.45 | -775.85 | -1,038.83 | -903.99 |
| Akaike Inf. Crit. | 1,958.84 | 2,130.63 | 1,842.91 | 1,575.71 | 2,101.66 | 1,831.99 |
|  | | | | | | |
| *Note:* | ^*^p<0.1; ^**^p<0.05; ^***^p<0.01 | | | | | |
|  | Standard errors clustered by village. Baseline district: Bogra. (n/y) – (no/yes), (m) - (meters). | | | | | |

Table S 20: Influence of binary flood and erosion impact on whether a respondent migrated or shifted their house location between wave 1 and wave 2 (linear regressions).

|  | | | | | | |
| --- | --- | --- | --- | --- | --- | --- |
|  | *Dependent variable:* | | | | | |
|  |  | | | | | |
|  | Migrated or shifted between w1 and w2? (n/y) | | | | | |
|  | (1) | (2) | (3) | (4) | (5) | (6) |
|  | | | | | | |
| Erosion impact (n/y) | 0.34^***^ | 0.32^***^ | 0.34^***^ | 0.30^***^ | 0.29^***^ | 0.28^***^ |
|  | (0.06) | (0.03) | (0.02) | (0.03) | (0.02) | (0.02) |
|  |  |  |  |  |  |  |
| Flood impact (n/y) | 0.06^**^ | 0.08^***^ | 0.06^**^ | 0.05^**^ | 0.06^**^ | 0.05^**^ |
|  | (0.03) | (0.03) | (0.02) | (0.02) | (0.03) | (0.02) |
|  |  |  |  |  |  |  |
| Aspirations (n/y) |  |  |  | 0.04 | 0.04^*^ | 0.06^**^ |
|  |  |  |  | (0.02) | (0.02) | (0.02) |
|  |  |  |  |  |  |  |
| Socio-econ. status |  |  |  | -0.04^***^ | -0.04^***^ | -0.04^***^ |
|  |  |  |  | (0.01) | (0.01) | (0.01) |
|  |  |  |  |  |  |  |
| Sex (f/m) |  |  |  | 0.17^***^ | 0.18^***^ | 0.15^***^ |
|  |  |  |  | (0.04) | (0.05) | (0.05) |
|  |  |  |  |  |  |  |
| Married? (n/y) |  |  |  | -0.03 | -0.05 | -0.01 |
|  |  |  |  | (0.05) | (0.05) | (0.05) |
|  |  |  |  |  |  |  |
| Age: 31-40 yr |  |  |  | -0.03 | -0.08^*^ | -0.05 |
|  |  |  |  | (0.04) | (0.04) | (0.04) |
|  |  |  |  |  |  |  |
| Age: 41-50 yr |  |  |  | -0.07^*^ | -0.13^***^ | -0.11^**^ |
|  |  |  |  | (0.04) | (0.04) | (0.04) |
|  |  |  |  |  |  |  |
| Age: 51-60 yr |  |  |  | -0.17^***^ | -0.23^***^ | -0.18^***^ |
|  |  |  |  | (0.04) | (0.05) | (0.04) |
|  |  |  |  |  |  |  |
| Age: 61+ yr |  |  |  | -0.15^***^ | -0.20^***^ | -0.19^***^ |
|  |  |  |  | (0.04) | (0.05) | (0.05) |
|  |  |  |  |  |  |  |
| Educ: primary |  |  |  | -0.01 | -0.02 | 0.0001 |
|  |  |  |  | (0.03) | (0.04) | (0.04) |
|  |  |  |  |  |  |  |
| Educ: secondary |  |  |  | -0.01 | -0.02 | -0.03 |
|  |  |  |  | (0.04) | (0.04) | (0.04) |
|  |  |  |  |  |  |  |
| Educ: SSC passed |  |  |  | 0.02 | 0.02 | -0.02 |
|  |  |  |  | (0.07) | (0.07) | (0.07) |
|  |  |  |  |  |  |  |
| Educ: HSC passed |  |  |  | -0.01 | -0.04 | -0.08 |
|  |  |  |  | (0.06) | (0.07) | (0.07) |
|  |  |  |  |  |  |  |
| Educ: university |  |  |  | -0.05 | -0.12^*^ | -0.08 |
|  |  |  |  | (0.06) | (0.07) | (0.07) |
|  |  |  |  |  |  |  |
| Income env.-dep.? (n/y) |  |  |  | 0.02 | 0.02 | 0.02 |
|  |  |  |  | (0.03) | (0.03) | (0.03) |
|  |  |  |  |  |  |  |
| Attachment (1-5) |  |  |  | 0.01 | 0.02 | 0.02 |
|  |  |  |  | (0.02) | (0.02) | (0.02) |
|  |  |  |  |  |  |  |
| Risk pref. (1-5) |  |  |  | 0.01 | 0.001 | -0.01 |
|  |  |  |  | (0.01) | (0.01) | (0.01) |
|  |  |  |  |  |  |  |
| Distance to river (m) |  |  |  | -0.001^***^ | -0.001^***^ | -0.001^***^ |
|  |  |  |  | (0.0001) | (0.0002) | (0.0001) |
|  |  |  |  |  |  |  |
| District: Gaibandha |  |  |  | -0.07 | -0.13 | -0.09 |
|  |  |  |  | (0.07) | (0.08) | (0.08) |
|  |  |  |  |  |  |  |
| District: Jamalpur |  |  |  | -0.03 | -0.04 | -0.03 |
|  |  |  |  | (0.06) | (0.06) | (0.06) |
|  |  |  |  |  |  |  |
| District: Kurigram |  |  |  | 0.04 | 0.02 | 0.003 |
|  |  |  |  | (0.04) | (0.05) | (0.04) |
|  |  |  |  |  |  |  |
| District: Manikganj |  |  |  | -0.06 | -0.11 | -0.10 |
|  |  |  |  | (0.05) | (0.07) | (0.06) |
|  |  |  |  |  |  |  |
| District: Sirajganj |  |  |  | 0.02 | 0.03 | 0.04 |
|  |  |  |  | (0.05) | (0.05) | (0.05) |
|  |  |  |  |  |  |  |
| District: Tangail |  |  |  | 0.18^***^ | 0.20^***^ | 0.23^***^ |
|  |  |  |  | (0.05) | (0.05) | (0.05) |
|  |  |  |  |  |  |  |
| Intercept | 0.11^***^ | 0.13^***^ | 0.12^***^ | 0.02 | 0.12 | 0.13 |
|  | (0.02) | (0.03) | (0.02) | (0.10) | (0.11) | (0.11) |
|  |  |  |  |  |  |  |
|  | | | | | | |
| Controls | No | No | No | Yes | Yes | Yes |
| Weights | No | Erosion | Flood | No | Erosion | Flood |
| Mean erosion control group | 0.13 | 0.14 | 0.14 | 0.13 | 0.14 | 0.14 |
| SD erosion control group | 0.34 | 0.35 | 0.35 | 0.34 | 0.35 | 0.35 |
| Observations | 1,595 | 1,271 | 1,271 | 1,271 | 1,271 | 1,271 |
| Log Likelihood | -797.56 | -999.88 | -843.61 | -597.83 | -919.76 | -760.63 |
| Akaike Inf. Crit. | 1,601.11 | 2,005.77 | 1,693.21 | 1,247.66 | 1,891.52 | 1,573.26 |
|  | | | | | | |
| *Note:* | ^*^p<0.1; ^**^p<0.05; ^***^p<0.01 | | | | | |
|  | Standard errors clustered by village. Baseline age: 18-30 yr. Baseline education: no education. Baseline district: Bogra. (n/y) – (no/yes), (f/m) – (female/male), (m) - (meters). | | | | | |

E.4 Different specifications of environmental affectedness (independent variable)

This study considers two types of environmental events: flooding and erosion. Since the two are not fully independent (erosion and floods occur both during the rainy monsoon season), including both events in the regression models might confound the results. Additional specifications include only erosion affectedness, only flood affectedness, or a combined indicator taking a value of 1 if the respondent has been affected by either erosion or floods, and 0 otherwise. The effects of erosion affectedness are substantively comparable to those presented in Table S 10 (Table S 21). Flood affetedness exerts a significant influence in those model specifications without controls, but substantively smaller than that of erosion affectedness (Table S 22). Lastly, the combined indicator of both erosion and flood affectedness exhibits a significant influence across model specifications, which is substantively similar to that of erosion alone (Table S 23).

To analyse whether the results of this study hold when employing not only respondents’ self-reported erosion affectedness, but an objective indicator of affectedness, I include the indicator of house loss as determined from satellite imagery (see Appendix A) into the main model (Table S 24). Objective house loss has a highly significant influence on the migration likelihood which is substantively comparable to that of self-reported strong erosion impact (see Table S 11). It hence appears appropriate to employ self-reported impacts in the main analyses.

Table S 21: Influence of binary erosion impact on whether a respondent migrated between wave 1 and wave 2 (linear regressions).

|  | | | | |
| --- | --- | --- | --- | --- |
|  | *Dependent variable:* | | | |
|  |  | | | |
|  | Migrated between w1 and w2? (n/y) | | | |
|  | (1) | (2) | (3) | (4) |
|  | | | | |
| Erosion impact (n/y) | 0.15^***^ | 0.13^***^ | 0.15^***^ | 0.14^***^ |
|  | (0.03) | (0.02) | (0.02) | (0.02) |
|  |  |  |  |  |
| Aspirations (n/y) |  |  | 0.03 | 0.03 |
|  |  |  | (0.02) | (0.02) |
|  |  |  |  |  |
| Socio-econ. status |  |  | -0.02^**^ | -0.02^*^ |
|  |  |  | (0.01) | (0.01) |
|  |  |  |  |  |
| Sex (f/m) |  |  | 0.14^***^ | 0.14^***^ |
|  |  |  | (0.04) | (0.04) |
|  |  |  |  |  |
| Married? (n/y) |  |  | -0.002 | -0.02 |
|  |  |  | (0.05) | (0.05) |
|  |  |  |  |  |
| Age: 31-40 yr |  |  | -0.03 | -0.05 |
|  |  |  | (0.04) | (0.04) |
|  |  |  |  |  |
| Age: 41-50 yr |  |  | -0.07^*^ | -0.12^***^ |
|  |  |  | (0.04) | (0.04) |
|  |  |  |  |  |
| Age: 51-60 yr |  |  | -0.15^***^ | -0.21^***^ |
|  |  |  | (0.04) | (0.04) |
|  |  |  |  |  |
| Age: 61+ yr |  |  | -0.17^***^ | -0.23^***^ |
|  |  |  | (0.04) | (0.04) |
|  |  |  |  |  |
| Educ: primary |  |  | 0.01 | 0.01 |
|  |  |  | (0.03) | (0.03) |
|  |  |  |  |  |
| Educ: secondary |  |  | 0.03 | 0.03 |
|  |  |  | (0.03) | (0.04) |
|  |  |  |  |  |
| Educ: SSC passed |  |  | -0.05 | -0.12^*^ |
|  |  |  | (0.06) | (0.07) |
|  |  |  |  |  |
| Educ: HSC passed |  |  | 0.04 | 0.03 |
|  |  |  | (0.06) | (0.06) |
|  |  |  |  |  |
| Educ: university |  |  | 0.02 | -0.01 |
|  |  |  | (0.06) | (0.06) |
|  |  |  |  |  |
| Income env.-dep.? (n/y) |  |  | 0.04^*^ | 0.04^*^ |
|  |  |  | (0.02) | (0.03) |
|  |  |  |  |  |
| Attachment (1-5) |  |  | -0.002 | 0.003 |
|  |  |  | (0.01) | (0.01) |
|  |  |  |  |  |
| Risk pref. (1-5) |  |  | 0.001 | -0.01 |
|  |  |  | (0.01) | (0.01) |
|  |  |  |  |  |
| Distance to river (m) |  |  | -0.0002^*^ | -0.0001 |
|  |  |  | (0.0001) | (0.0002) |
|  |  |  |  |  |
| District: Gaibandha |  |  | -0.001 | 0.02 |
|  |  |  | (0.06) | (0.07) |
|  |  |  |  |  |
| District: Jamalpur |  |  | 0.04 | 0.06 |
|  |  |  | (0.05) | (0.06) |
|  |  |  |  |  |
| District: Kurigram |  |  | 0.09^**^ | 0.11^***^ |
|  |  |  | (0.04) | (0.04) |
|  |  |  |  |  |
| District: Manikganj |  |  | 0.005 | 0.01 |
|  |  |  | (0.05) | (0.06) |
|  |  |  |  |  |
| District: Sirajganj |  |  | 0.01 | 0.04 |
|  |  |  | (0.05) | (0.05) |
|  |  |  |  |  |
| District: Tangail |  |  | 0.03 | 0.03 |
|  |  |  | (0.04) | (0.04) |
|  |  |  |  |  |
| Intercept | 0.11^***^ | 0.14^***^ | 0.03 | 0.08 |
|  | (0.01) | (0.02) | (0.09) | (0.10) |
|  |  |  |  |  |
|  | | | | |
| Controls | No | No | Yes | Yes |
| Weights | No | Erosion | No | Erosion |
| Mean erosion control group | 0.11 | 0.11 | 0.11 | 0.11 |
| SD erosion control group | 0.31 | 0.32 | 0.31 | 0.32 |
| Observations | 1,595 | 1,271 | 1,271 | 1,271 |
| Log Likelihood | -607.65 | -848.88 | -479.29 | -796.39 |
| Akaike Inf. Crit. | 1,219.31 | 1,701.76 | 1,008.58 | 1,642.77 |
|  | | | | |
| *Note:* | ^*^p<0.1; ^**^p<0.05; ^***^p<0.01 | | | |
|  | Standard errors clustered by village. Baseline age: 18-30 yr. Baseline education: no education. Baseline district: Bogra. (n/y) – (no/yes), (f/m) – (female/male), (m) - (meters). | | | |

Table S 22: Influence of binary flood impact on whether a respondent migrated between wave 1 and wave 2 (linear regressions).

|  | | | | |
| --- | --- | --- | --- | --- |
|  | *Dependent variable:* | | | |
|  |  | | | |
|  | Migrated between w1 and w2? (n/y) | | | |
|  | (1) | (2) | (3) | (4) |
|  | | | | |
| Flood impact (n/y) | 0.07^**^ | 0.02 | 0.07^***^ | 0.01 |
|  | (0.03) | (0.02) | (0.02) | (0.02) |
|  |  |  |  |  |
| Aspirations (n/y) |  |  | 0.03 | 0.04^*^ |
|  |  |  | (0.02) | (0.02) |
|  |  |  |  |  |
| Socio-econ. status |  |  | -0.02^*^ | -0.005 |
|  |  |  | (0.01) | (0.01) |
|  |  |  |  |  |
| Sex (f/m) |  |  | 0.14^***^ | 0.13^***^ |
|  |  |  | (0.04) | (0.05) |
|  |  |  |  |  |
| Married? (n/y) |  |  | -0.02 | 0.0004 |
|  |  |  | (0.05) | (0.05) |
|  |  |  |  |  |
| Age: 31-40 yr |  |  | -0.04 | -0.04 |
|  |  |  | (0.04) | (0.04) |
|  |  |  |  |  |
| Age: 41-50 yr |  |  | -0.08^**^ | -0.10^**^ |
|  |  |  | (0.04) | (0.04) |
|  |  |  |  |  |
| Age: 51-60 yr |  |  | -0.16^***^ | -0.15^***^ |
|  |  |  | (0.04) | (0.04) |
|  |  |  |  |  |
| Age: 61+ yr |  |  | -0.19^***^ | -0.20^***^ |
|  |  |  | (0.04) | (0.04) |
|  |  |  |  |  |
| Educ: primary |  |  | 0.01 | 0.002 |
|  |  |  | (0.03) | (0.03) |
|  |  |  |  |  |
| Educ: secondary |  |  | 0.02 | 0.03 |
|  |  |  | (0.03) | (0.04) |
|  |  |  |  |  |
| Educ: SSC passed |  |  | -0.06 | -0.11 |
|  |  |  | (0.06) | (0.07) |
|  |  |  |  |  |
| Educ: HSC passed |  |  | 0.05 | 0.02 |
|  |  |  | (0.06) | (0.06) |
|  |  |  |  |  |
| Educ: university |  |  | 0.01 | 0.02 |
|  |  |  | (0.06) | (0.07) |
|  |  |  |  |  |
| Income env.-dep.? (n/y) |  |  | 0.04^*^ | 0.05^*^ |
|  |  |  | (0.02) | (0.03) |
|  |  |  |  |  |
| Attachment (1-5) |  |  | -0.004 | -0.01 |
|  |  |  | (0.01) | (0.02) |
|  |  |  |  |  |
| Risk pref. (1-5) |  |  | 0.002 | -0.01 |
|  |  |  | (0.01) | (0.01) |
|  |  |  |  |  |
| Distance to river (m) |  |  | -0.0003^**^ | -0.0004^***^ |
|  |  |  | (0.0001) | (0.0001) |
|  |  |  |  |  |
| District: Gaibandha |  |  | -0.02 | -0.06 |
|  |  |  | (0.06) | (0.07) |
|  |  |  |  |  |
| District: Jamalpur |  |  | 0.03 | 0.03 |
|  |  |  | (0.05) | (0.06) |
|  |  |  |  |  |
| District: Kurigram |  |  | 0.06^*^ | 0.02 |
|  |  |  | (0.04) | (0.04) |
|  |  |  |  |  |
| District: Manikganj |  |  | -0.02 | -0.06 |
|  |  |  | (0.05) | (0.06) |
|  |  |  |  |  |
| District: Sirajganj |  |  | 0.0003 | -0.04 |
|  |  |  | (0.05) | (0.05) |
|  |  |  |  |  |
| District: Tangail |  |  | 0.03 | 0.04 |
|  |  |  | (0.04) | (0.05) |
|  |  |  |  |  |
| Intercept | 0.12^***^ | 0.19^***^ | 0.10 | 0.25^**^ |
|  | (0.01) | (0.02) | (0.09) | (0.10) |
|  |  |  |  |  |
|  | | | | |
| Controls | No | No | Yes | Yes |
| Weights | No | Flood | No | Flood |
| Mean erosion control group | 0.11 | 0.11 | 0.11 | 0.11 |
| SD erosion control group | 0.31 | 0.32 | 0.31 | 0.32 |
| Observations | 1,595 | 1,271 | 1,271 | 1,271 |
| Log Likelihood | -629.65 | -724.60 | -497.54 | -687.61 |
| Akaike Inf. Crit. | 1,263.31 | 1,453.20 | 1,045.07 | 1,425.21 |
|  | | | | |
| *Note:* | ^*^p<0.1; ^**^p<0.05; ^***^p<0.01 | | | |
|  | Standard errors clustered by village. Baseline age: 18-30 yr. Baseline education: no education. Baseline district: Bogra. (n/y) – (no/yes), (f/m) – (female/male), (m) - (meters). | | | |

Table S 23: Influence of binary, combined erosion or flood impact on whether a respondent migrated between wave 1 and wave 2 (linear regressions). The variable “Erosion or flood impact” is 1 if the respondent has been affected by either erosion of floods in the previous year.

|  | | | | |
| --- | --- | --- | --- | --- |
|  | *Dependent variable:* | | | |
|  |  | | | |
|  | Migrated between w1 and w2? (n/y) | | | |
|  | (1) | (2) | (3) | (4) |
|  | | | | |
| Erosion or flood impact (n/y) | 0.11^***^ | 0.10^***^ | 0.10^***^ | 0.10^***^ |
|  | (0.02) | (0.02) | (0.03) | (0.02) |
|  |  |  |  |  |
| Aspirations (n/y) |  |  | 0.03 | 0.02 |
|  |  |  | (0.02) | (0.02) |
|  |  |  |  |  |
| Socio-econ. status |  |  | -0.02^*^ | -0.02^***^ |
|  |  |  | (0.01) | (0.01) |
|  |  |  |  |  |
| Sex (f/m) |  |  | 0.14^***^ | 0.11^***^ |
|  |  |  | (0.04) | (0.04) |
|  |  |  |  |  |
| Married? (n/y) |  |  | -0.02 | 0.01 |
|  |  |  | (0.05) | (0.04) |
|  |  |  |  |  |
| Age: 31-40 yr |  |  | -0.04 | 0.02 |
|  |  |  | (0.04) | (0.03) |
|  |  |  |  |  |
| Age: 41-50 yr |  |  | -0.08^**^ | -0.02 |
|  |  |  | (0.04) | (0.03) |
|  |  |  |  |  |
| Age: 51-60 yr |  |  | -0.16^***^ | -0.07^**^ |
|  |  |  | (0.04) | (0.04) |
|  |  |  |  |  |
| Age: 61+ yr |  |  | -0.18^***^ | -0.10^***^ |
|  |  |  | (0.04) | (0.04) |
|  |  |  |  |  |
| Educ: primary |  |  | -0.002 | -0.01 |
|  |  |  | (0.03) | (0.03) |
|  |  |  |  |  |
| Educ: secondary |  |  | 0.02 | 0.04 |
|  |  |  | (0.03) | (0.03) |
|  |  |  |  |  |
| Educ: SSC passed |  |  | -0.07 | -0.05 |
|  |  |  | (0.06) | (0.06) |
|  |  |  |  |  |
| Educ: HSC passed |  |  | 0.04 | 0.13^***^ |
|  |  |  | (0.06) | (0.05) |
|  |  |  |  |  |
| Educ: university |  |  | -0.0003 | 0.08 |
|  |  |  | (0.06) | (0.05) |
|  |  |  |  |  |
| Income env.-dep.? (n/y) |  |  | 0.05^*^ | 0.02 |
|  |  |  | (0.02) | (0.02) |
|  |  |  |  |  |
| Attachment (1-5) |  |  | -0.01 | 0.005 |
|  |  |  | (0.01) | (0.01) |
|  |  |  |  |  |
| Risk pref. (1-5) |  |  | 0.001 | 0.01^*^ |
|  |  |  | (0.01) | (0.01) |
|  |  |  |  |  |
| Distance to river (m) |  |  | -0.0003^***^ | -0.0001 |
|  |  |  | (0.0001) | (0.0001) |
|  |  |  |  |  |
| District: Gaibandha |  |  | -0.01 | 0.01 |
|  |  |  | (0.06) | (0.05) |
|  |  |  |  |  |
| District: Jamalpur |  |  | 0.04 | 0.03 |
|  |  |  | (0.05) | (0.05) |
|  |  |  |  |  |
| District: Kurigram |  |  | 0.06 | 0.08^**^ |
|  |  |  | (0.04) | (0.04) |
|  |  |  |  |  |
| District: Manikganj |  |  | -0.03 | 0.01 |
|  |  |  | (0.05) | (0.05) |
|  |  |  |  |  |
| District: Sirajganj |  |  | -0.001 | 0.01 |
|  |  |  | (0.05) | (0.05) |
|  |  |  |  |  |
| District: Tangail |  |  | 0.04 | 0.04 |
|  |  |  | (0.04) | (0.04) |
|  |  |  |  |  |
| Intercept | 0.06^***^ | 0.08^***^ | 0.05 | -0.14 |
|  | (0.02) | (0.01) | (0.09) | (0.08) |
|  |  |  |  |  |
|  | | | | |
| Controls | No | No | Yes | Yes |
| Weights | No | Erosion/flood | No | Erosion/flood |
| Mean erosion control group | 0.11 | 0.11 | 0.11 | 0.11 |
| SD erosion control group | 0.31 | 0.32 | 0.31 | 0.32 |
| Observations | 1,595 | 1,271 | 1,271 | 1,271 |
| Log Likelihood | -626.30 | -577.42 | -496.60 | -538.83 |
| Akaike Inf. Crit. | 1,256.60 | 1,158.84 | 1,043.19 | 1,127.67 |
|  | | | | |
| *Note:* | ^*^p<0.1; ^**^p<0.05; ^***^p<0.01 | | | |
|  | Standard errors clustered by village. Baseline age: 18-30 yr. Baseline education: no education. Baseline district: Bogra. (n/y) – (no/yes), (f/m) – (female/male), (m) - (meters). | | | |

Table S 24: Influence of objective erosion impact on whether a respondent migrated between wave 1 and wave 2 (linear regressions; reduced form not showing control variables – full table available upon request).

|  | | | | |
| --- | --- | --- | --- | --- |
|  | *Dependent variable:* | | | |
|  |  | | | |
|  | Migrated between w1 and w2? (n/y) | | | |
|  | (1) | (2) | (3) | (4) |
|  | | | | |
| House lost (obj) | 0.20^***^ | 0.24^***^ | 0.23^***^ | 0.22^***^ |
|  | (0.07) | (0.03) | (0.04) | (0.03) |
|  |  |  |  |  |
| Else: strong erosion impact (subj) | 0.25^***^ | 0.28^***^ | 0.25^***^ | 0.26^***^ |
|  | (0.06) | (0.06) | (0.05) | (0.06) |
|  |  |  |  |  |
| Else: medium erosion impact (subj) | 0.09^***^ | 0.15^***^ | 0.10^***^ | 0.14^***^ |
|  | (0.03) | (0.04) | (0.03) | (0.04) |
|  |  |  |  |  |
| Else: some erosion impact (subj) | 0.04 | -0.003 | 0.03 | -0.08 |
|  | (0.03) | (0.07) | (0.06) | (0.07) |
|  |  |  |  |  |
| Flood affected | 0.02 | -0.08^***^ | 0.03 | -0.08^***^ |
|  | (0.03) | (0.03) | (0.02) | (0.03) |
|  |  |  |  |  |
|  | | | | |
| Controls | No | No | Yes | Yes |
| Weights | No | Erosion obj | No | Erosion obj |
| Mean erosion control group | 0.11 | 0.11 | 0.11 | 0.11 |
| SD erosion control group | 0.31 | 0.32 | 0.31 | 0.32 |
| Observations | 1,595 | 1,271 | 1,271 | 1,271 |
| Log Likelihood | -595.67 | -2,025.33 | -468.87 | -1,924.95 |
| Akaike Inf. Crit. | 1,203.35 | 4,062.65 | 995.74 | 3,907.91 |
|  | | | | |
| *Note:* | ^*^p<0.1; ^**^p<0.05; ^***^p<0.01 | | | |
|  | Standard errors clustered by village. Baseline erosion impact: no impact. (n/y) – (no/yes), (m) - (meters). | | | |

Appendix F: Pre-registration

F.1 Anonymized version of pre-analysis plan

Even if two individuals or households are exposed to the same environmental/climatic event, they do not necessarily react the same way: One might migrate, while the other might stay. In this project, we examine the micro-level processes connecting the exposure to an environmental event and a migration decision. Individuals will only migrate if they have both an aspiration and the ability to move (J⊘rgen Carling, 2002; Jørgen Carling & Schewel, 2018). Conversely, this implies that there might be individuals who would like to migrate, but cannot (involuntary non-migrants or trapped populations) as well as others who could migrate, but do not want to, even though they might face a physical need to move (voluntary non-migrants). This project seeks to distinguish these three groups (migrants being the third group) by examining the relationship of migration aspirations and the ability to move.

Thereby, we define “migration aspirations” as “a person’s thoughts and feelings about the prospect of changing their place of residence” (Jørgen Carling, 2019, p. 8). We differentiate these subjective aspirations from an objective “need to move”, which we define as a necessity to move since an individual’s/household’s livelihood comes under pressure, since it is no longer sustainable in the current location. Differentiating aspirations and need is important to fully understand micro level decision processes. Black et al. (2013), for instance, only consider vulnerability to extreme events and ability to move to infer the (conceptual) existence of trapped populations. This concept lacks, however, the individuals’ aspirations, which are crucial for the definition of trapped populations. Therefore, we need to understand what contributes to migration aspirations:

*RQ1: How are migration aspirations influenced by exposure to an environmental event and how do they relate to actual migration moves?*

Migration aspirations are influenced by a number of factors, which we include as control variables into the following analyses. One important factor is an individual’s attachment to place. Adams and Adger (2013) argue that environmental factors enter the migration decision-making process through their contribution to place utility, which they define as a function of both affective and instrumental bonds to location. Further, Adams (2016) shows that place attachment can have a stronger effect on immobility than resource constraints. Other mediating factors that we will include are faith in God (where we expect more religious individuals to have a lower desire to move since they perceive reduced agency over their life) and risk preference (where we expect risk averse individuals to have a lower desire to move since the risk of moving is perceived as higher than the risk of staying, following Carling (J⊘rgen Carling, 2002)).

Following Black et al. (2013), we proxy a household’s need to move by their vulnerability to a certain environmental event. Vulnerability is formed by three components, namely exposure, sensitivity and adaptive capacity (IPCC, 2007). We define exposure as the perceived or actual (direct or indirect) impact of environmental change on the livelihood of the household. Sensitivity is the degree to which a system is affected by or responsive to environmental stimuli, whereas adaptive capacitiy describes the ability to prepare for, respond to and tackle the effects of environmental change. Since in our research design, exogenous variation occurs only in terms of exposure, this is our independent variable for the subsequent hypotheses (alternatively, we might use proximity to the riverbank as an instrument), whereas we take sensitivity and adaptive capacities into account by controlling for them.

We plan to measure migration aspirations in each of the four rounds of the panel. In the following, we describe our hypotheses and related analyses for two rounds for reasons of simplicity. The reasoning can, however, be extended to all four rounds. In the baseline survey before the monsoon, we measure migration aspirations for all respondents. During the monsoon, a part of the sample will be exposed to environmental events (mainly riverbank erosion and/or floods), while the other part will not be exposed. Further, a part of the sample will migrate during or after the monsoon (either as an entire household or individually), while the other part will not migrate. When we conduct the second round of interviews after the monsoon, we are thus faced with four groups (Table S 25).

Table S 25: Respondents of the first follow-up survey fall into one of four categories.

|  | Has migrated | Has not migrated |
| --- | --- | --- |
| Has been exposed | 1 | 2 |
| Has not been exposed | 3 | 4 |

These four groups allow for different analyses related to migration aspirations. First, we will compare those who have migrated (groups 1+3) with those who have not migrated (groups 2+4) in terms of their migration aspirations during the baseline survey. Tjaden, Auer, and Laczko (2019) have shown a strong association between migration intentions and actual migration flows. If this holds true, measuring migration aspirations could serve as a proxy to predict migration flows. Therefore, we test the following hypothesis:

*H1: The higher the migration aspiration before the monsoon, the higher the likelihood of migrating during or after the monsoon, controlling for ability to move.*

Second, we will re-assess the migration aspirations after the monsoon for those who have not migrated (groups 2+4). Exposure corresponds to a livelihood threatening loss of assets or land. Therefore, we expect those who have been exposed to an environmental event (group 2) to have higher migration aspirations after the monsoon season than before. Further, migration aspirations are not a stable character trait, but are highly situational and can be volatile (Jørgen Carling, 2019, p. 14). We would expect the overall livelihood situation in the study area to be more challenging immediately after the monsoon (e.g. due to flood-related infrastructure damages or the exposure of close relatives or friends, even if the own household has not been exposed). Accordingly, we expect migration aspirations to be higher after the monsoon than before also for those respondents who have not been exposed themselves. We thus expect an overall increase in migration aspirations among all respondents who have not migrated, but this increase should be stronger among those who have been exposed than among those who have not been exposed. We plan to analyze this effect in a difference-in-difference design.

*H2: Average migration aspirations are higher after than before the monsoon.*

*H3: Migration aspirations increase more for respondents who have been exposed than for those who have not.*

Third, we will focus in more detail on the characterization of those who have not migrated (groups 2+4). It is important to differentiate whether their non-migration is because they have no migration aspirations in the first place (voluntary non-migrants), or because they have aspirations, but lack the ability to move (involuntary non-migrants): If households do not have enough resources to migrate, they will stay *in situ* despite potential migration aspirations. This is the basic idea behind the term “trapped populations”. While the term has seen a lot of conceptual discussion in recent literature (cf. Ayeb-Karlsson et al., 2018 for a discursive review), little empirical work has been done investigating the extent and nature of trapped populations:

*RQ2: How many of those who did not migrate are voluntary non-migrants, and how many are involuntary non-migrants?*

Irrespective of whether they are voluntary or involuntary non-migrants, those who have been exposed (group 2) have to cope with the effect of the exposure on their livelihood *in situ*, given that they have not migrated. If policy makers wish to support these parts of the population, they need to understand these coping strategies. Also, they need to understand the obstacles preventing those who have migration aspirations from actually moving. Therefore, we ask the exploratory questions:

*RQ3: What are in situ coping strategies of those respondents who are exposed to environmental change, but do not migrate?*

*RQ4: Which factors prevent involuntary non-migrants from migrating?*

Also, policy makers require information on the socio-demographic composition of the three groups (migrants as well as voluntary and non-voluntary non-migrants). The ability to move is correlated to a household’s access to resources, which is, for instance, lower for poor and female headed households (Akter et al., 2019; G. M. M. Alam, 2017).

*RQ5: What is the socio-demographic composition of migrants, voluntary non-migrants and involuntary non-migrants, differentiated by exposure?*

To analyze these questions, we mostly use data from the household surveys. Specifically, we will use the following constructs:

- Exposure – determined from the perception of the environmental event and/or the affectedness by the event (depending on the results of section XX).
- Sensitivity – determined from occupation, land/house ownership, financial resources (income, credit, savings, and meals per day) as well as the diversity of livelihood strategies.
- Adaptive capacities – determined from adaptation strategies applied for past erosion/flood events, education (as a proxy for human capital following van der Land and Hummel (van der Land & Hummel, 2013)), wealth (as a proxy for financial capital) and social networks.
- Attachment to place – determined from length of residence in present location, home ownership, and a self-assessment.
- Faith in god – determined from questions about religious practices
- Risk preference – determined from a self-assessment
- Desire to move – determined from present aspirations to move, their choice in a hypothetical decision scenario, and whether they had thought about migrating in the past, following Carling and Schewel (2018) and Carling (2019).
- Ability to move – determined from the respondents’ financial resources (physical capital) as well as from their social network and whether they or other members of the household have previously migrated (social capital), following Wiederkehr et al. (2019, p. 7).
- Migration decisions – derived from the post-monsoon survey as well as from tracking the respondents during the monsoon season.

F.2 Modifications to the pre-analysis plan

I partially adapted the pre-analysis plan and report the differences here for reasons of transparency.

- Scope 1: Initially, this subproject was conceived to study both the link from environmental exposure to migration aspirations, and from aspirations to actual moves. To narrow the focus of the paper, it focuses only on the link from environmental exposure to actual moves, controlling for a person’s migration aspirations. The link from environmental exposure to migration aspirations is examined in another subproject / paper.
- Scope 2: Research question 2 (existence of trapped populations) is likewise studied in a separate project.

References

Adams, H. (2016). Why populations persist: mobility, place attachment and climate change. *Population and Environment*, *37*(4), 429–448. https://doi.org/10.1007/s11111-015-0246-3

Adams, H., & Adger, N. W. (2013). The contribution of ecosystem services to place utility as a determinant of migration decision-making. *Environmental Research Letters*, *8*(1), 15006. https://doi.org/10.1088/1748-9326/8/1/015006

Akter, K., Dey, S., & Hasan, S. (2019). Riverbank erosion and its impact on rural women: Case study of Ulania village in Bangladesh. *Asian Journal of Women's Studies*, *25*(1), 76–95. https://doi.org/10.1080/12259276.2019.1577343

Alam, G. M. M. (2017). Livelihood Cycle and Vulnerability of Rural Households to Climate Change and Hazards in Bangladesh. *Environmental Management*, *59*(5), 777–791. https://doi.org/10.1007/s00267-017-0826-3

Alam, G. M. M., Alam, K., Mushtaq, S., & Clarke, M. L. (2017). Vulnerability to climatic change in riparian char and river-bank households in Bangladesh: Implication for policy, livelihoods and social development. *Ecological Indicators*, *72*, 23–32. https://doi.org/10.1016/j.ecolind.2016.06.045

Ayeb-Karlsson, S., Smith, C. D., & Kniveton, D. (2018). A discursive review of the textual use of ‘trapped’ in environmental migration studies: The conceptual birth and troubled teenage years of trapped populations. *Ambio*, *47*(5), 557–573. https://doi.org/10.1007/s13280-017-1007-6

Black, R., Arnell, N. W., Adger, W. N., Thomas, D., & Geddes, A. (2013). Migration, immobility and displacement outcomes following extreme events. *Environmental Science & Policy*, *27*, S32-S43. https://doi.org/10.1016/j.envsci.2012.09.001

Carling, J [J⊘rgen] (2002). Migration in the age of involuntary immobility: Theoretical reflections and Cape Verdean experiences. *Journal of Ethnic and Migration Studies*, *28*(1), 5–42. https://doi.org/10.1080/13691830120103912

Carling, J [Jørgen]. (2019). *Measuring migration aspirations and related concepts: MIGNEX Background Paper*. Oslo. Peace Research Institute Oslo.

Carling, J [Jørgen], & Schewel, K. (2018). Revisiting aspiration and ability in international migration. *Journal of Ethnic and Migration Studies*, *44*(6), 945–963. https://doi.org/10.1080/1369183X.2017.1384146

Freihardt, J., & Frey, O. (2023). Assessing riverbank erosion in Bangladesh using time series of Sentinel-1 radar imagery in the Google Earth Engine. *Natural Hazards and Earth System Science*, *23*(2), 751–770. https://doi.org/10.5194/nhess-23-751-2023

Gray, C. L., & Mueller, V. (2012). Natural disasters and population mobility in Bangladesh. *Proceedings of the National Academy of Sciences*, *109*(16), 6000–6005. https://doi.org/10.1073/pnas.1115944109

Hainmueller, J. (2012). Entropy balancing for causal effects: A multivariate reweighting method to produce balanced samples in observational studies. *Political Analysis*, *20*(1), 25–46.

Horrace, W. C., & Oaxaca, R. L. (2006). Results on the bias and inconsistency of ordinary least squares for the linear probability model. *Economics Letters*, *90*(3), 321–327. https://doi.org/10.1016/j.econlet.2005.08.024

IPCC. (2007). *Climate change 2007 - impacts, adaptation and vulnerability: Contribution of Working Group II to the Fourth Assessment Report of the Intergovernmental Panel on Climate Change* (1. publ). Cambridge Univ. Press. http://www.ipcc.ch/ipccreports/ar4-wg2.htm

Tjaden, J., Auer, D., & Laczko, F. (2019). Linking Migration Intentions with Flows: Evidence and Potential Use. *International Migration*, *57*(1), 36–57. https://doi.org/10.1111/imig.12502

van der Land, V., & Hummel, D. (2013). Vulnerability and the Role of Education in Environmentally Induced Migration in Mali and Senegal. *Ecology and Society*, *18*(4). https://doi.org/10.5751/ES-05830-180414

Wiederkehr, C., Schröter, M., Adams, H., Seppelt, R., & Hermans, K. (2019). How does nature contribute to human mobility? A conceptual framework and qualitative analysis. *Ecology and Society*, *24*(4). https://doi.org/10.5751/ES-11318-240431
